# A Review of Geophysical Modeling Based on Particle Swarm Optimization

**DOI:** 10.1007/s10712-021-09638-4

**Published:** 2021-04-13

**Authors:** Francesca Pace, Alessandro Santilano, Alberto Godio

**Affiliations:** 1 Department of Environment, Land and Infrastructure Engineering (DIATI), Politecnico di Torino, Corso Duca degli Abruzzi 24, 10129 Turin, Italy; 2 Institute of Geosciences and Earth Resources - National Research Council (IGG-CNR), Via Moruzzi 1, 56124 Pisa, Italy

**Keywords:** Particle swarm optimization, Stochastic inverse modeling, Inversion, Swarm intelligence, Optimization, Joint optimization

## Abstract

This paper reviews the application of the algorithm particle swarm optimization (PSO) to perform stochastic inverse modeling of geophysical data. The main features of PSO are summarized, and the most important contributions in several geophysical fields are analyzed. The aim is to indicate the fundamental steps of the evolution of PSO methodologies that have been adopted to model the Earth’s subsurface and then to undertake a critical evaluation of their benefits and limitations. Original works have been selected from the existing geophysical literature to illustrate successful PSO applied to the interpretation of electromagnetic (magnetotelluric and time-domain) data, gravimetric and magnetic data, self-potential, direct current and seismic data. These case studies are critically described and compared. In addition, joint optimization of multiple geophysical data sets by means of multi-objective PSO is presented to highlight the advantage of using a single solver that deploys Pareto optimality to handle different data sets without conflicting solutions. Finally, we propose best practices for the implementation of a customized algorithm from scratch to perform stochastic inverse modeling of any kind of geophysical data sets for the benefit of PSO practitioners or inexperienced researchers.

**Article Highlights**

- Stochastic inverse modeling of geophysical data using the particle swarm optimization algorithm
- Best practices for the adoption of particle swarm optimization in geophysics
- Multi-objective optimization of geophysical data

## Introduction

The link between geophysical data and the properties of the Earth’s subsurface is provided by the modeling process, that is, forward and inverse modeling. These modeling procedures allow us to derive a subsurface model that interprets the observed data; the core of the procedure requires the inverse problem to be solved. This means solving a nonlinear, multi-parametric and ill-posed problem affected by the equivalence of solutions. Thus, there are many models that can equally fit the data within a given tolerance threshold (Tarantola 2005). The standard approach is the iterated and linearized inversion based on a local search of the model domain (Sen and Stoffa 2013). The main issues of this approach include firstly the choice of the reference model used to initialize the inversion, which can strongly bias the result and hence the interpretation, and secondly the inversion can find local rather than global solutions (i.e., the local minimum “syndrome”, Sen and Stoffa 2013).

To solve the nonlinear inverse problem, it is also possible to perform a global optimization instead of a linearized inversion. The global search approach, also called probabilistic or stochastic inverse modeling, is represented by methods like Monte Carlo or metaheuristics (Sen and Stoffa 2013). Global search methods have become of major interest in geophysics because they are theoretically able to find the global minimum of a function as the final solution without being trapped in one of several local minima. The main reason for this is that the model space is sampled either randomly or according to a specific strategy (e.g., adaptive behavior). Consequently, global search algorithms are time-consuming, while derivative-based algorithms converge after a few iterations. The essential advantage of global search algorithms is that the final solution is independent from the initial guess of the starting model. Unfortunately, the application of global search algorithms to geophysical inversion has been hindered by their high computational costs. However, the striking recent improvements in computer efficiency have enabled the decrease in the computer time required to run these algorithms.

The family of global search algorithms is divided into two main groups. The first one is represented by Monte Carlo (MC) methods and is based on the random sampling of the search space of the solutions (Sambridge and Mosegaard 2002). The second group encompasses the metaheuristic methods, such as nature-inspired algorithms. They are examples of Computational Intelligence algorithms based on biological systems. Among the metaheuristic methods, evolutionary computation (EC) models genetic and behavioral evolution, while swarm intelligence (SI) models the social behavior of organisms living in groups. EC and SI are referred to as population-based algorithms since they are based on the behavior of groups of individuals (Engelbrecht 2007). The EC tenet is that the individuals with the best chromosomes survive (and the weakest individuals have to die), so that only the selected chromosomes are inherited by the new generations. The most important example of an EC algorithm is the genetic algorithm (GA) (Engelbrecht 2007).

SI is instead the problem-solving behavior emerging from the interactions of agents in a group. SI mimics the naturally based social dynamics that provide individuals with more information than their own senses obtain. The algorithmic models of SI are referred to as *computational swarm intelligence* (CSI), whose main paradigms are *particle swarm optimization* (PSO) and *ant colony optimization* (ACO) (Kennedy et al. 2001). Many emerging real-world applications of EC and CSI are telecommunication networks, training of neural networks, game learning, clustering, design, bioinformatics, data mining (Engelbrecht 2007) and chemical engineering (Valadi and Siarry 2014).

GA, PSO and ACO are theoretically able to find the global minimum of the objective function without being trapped in one of the several local minima. This point is of pivotal importance in their application to geophysics because of the equivalence issue of the geophysical inverse problem. The most important metaheuristics applied to the inversion of geophysical data are simulated annealing (SA) (Kirkpatrick et al. 1983; Sen et al. 1993), GA (Sen and Stoffa 2013), ACO (Yuan et al. 2009), grey wolf optimizer (Agarwal et al. 2018) and PSO (Shaw and Srivastava 2007). To date, GA has been more widely adopted than PSO in geophysical modeling: 1-D seismic waveform inversion (Stoffa and Sen 1992), magnetotelluric (MT) data (Pérez-Flores and Schultz 2002; Everett and Schultz 1993) and reservoir modeling (Sen et al. 1995). However, some studies have demonstrated that PSO outperforms GA for accuracy and convergence in several geophysical applications (Yuan et al. 2009; Fernández Martínez et al. 2010a; Song et al. 2012; Pace et al. 2019b).

### General Applications of PSO

PSO has been successfully utilized in many fields, such as structural design (Perez and Behdinan 2007), solar photovoltaic systems (Khare and Rangnekar 2013), epidemic modeling of Sars-Cov-2 (Godio et al. 2020; Al-qaness et al. 2020), hydrogeology (Fernández Martínez et al. 2012), geotechnical engineering (Cheng et al. 2007; Armaghani et al. 2017; Yin et al. 2018), reservoir engineering (Ahmadi et al. 2013), computer vision (Jin et al. 2017), artificial neural networks (Song et al. 2007), biomedical engineering (modeling of the spread of antibiotic resistance) (Wachowiak et al. 2004), electronics and electromagnetics (Robinson and Rahmat-Samii 2004), power systems (AlRashidi and El-Hawary 2009), robotics (robot path planning), and signal processing (Poli 2008; Adhan and Bansal 2017 and references therein).

**Fig. 1:**
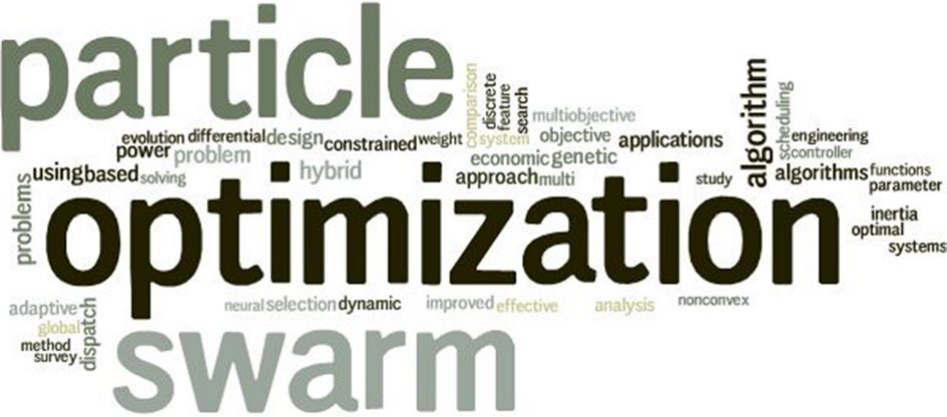
Word cloud from the titles of the most cited one-thousand papers about PSO published from 1995 to 2020. The data source is Google Scholar and encompasses journal articles, books and conference proceedings. Data analysis: Harzing (2007) Publish or Perish, available from https://harzing.com/resources/publish-or-perish

A clear overview of the most common PSO applications is graphically shown in Fig. 1. It is a word cloud generated from the one-thousand most cited PSO papers in Google Scholar between 1995 and 2020. The year 1995 is a milestone in the PSO literature since the first paper about PSO was published by Kennedy and Eberhart, which today counts more than 60,000 citations. During the same time span of 25 years, more than 15,000 works (journal articles, conference proceedings, books) that contain PSO in the title have been published according to Google Scholar. The overall H-index is 232. The mean citations per year are 4 and the mean author count is 3 (data analysis: Publish or Perish, available online; query date: July 2020).

**Fig. 2:**
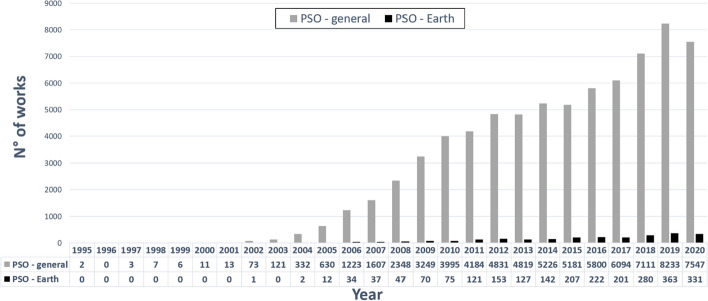
Number of works published in 1995–2020 including PSO in the title or abstract or keywords (data source: Scopus). Grey bins refer to all scientific fields, black bins refer to “Earth and planetary science”. The table below the horizontal axis counts the number of PSO-related works per year

According to Scopus, more than 73,000 works (journal articles, conference proceedings, books) were published between 1995 and 2020, which included PSO in the title or abstract or keywords. They are marked in the grey bins in Fig. 2 and classified by the year of publication. In the field of “Earth and planetary science” a total of 2458 works was published, as depicted in the black bins in Fig. 2. Clearly, there was an increasing linear trend in PSO-related works in this period. PSO has received growing attention in the field of geoscience as well, in particular near-surface applications for geological, hydrogeological and engineering studies, and large-scale geological and structural exploration of geo-resources.

### PSO in Geophysical Studies: An Overview

Even though recent reviews of PSO in engineering-related fields have been published (e.g., Khare and Rangnekar 2013), little attention has been paid to a comprehensive review of PSO applied to geosciences, despite demonstrating attractive features and successful geophysical applications. The fundamental theoretical basis of PSO can be found in Engelbrecht (2007), which deals with general applications of PSO to the optimization of benchmark mathematical functions. The book of Sen and Stoffa (2013) focuses on global search methods applied to the geophysical inversion. However, it extensively describes methods such as Monte Carlo, SA and GA, but devotes only a paragraph to PSO.

The earliest applications of PSO to geophysical data involved direct current (DC), induced polarization (IP) and MT (Shaw and Srivastava 2007). Subsequently, research studies improved the PSO algorithm by focusing on the tuning of the input parameters, stability region, convergence and PSO variants (Fernández Martínez et al. 2010a, b). This resulted in a better solution of the geophysical inverse problem. At the same time, PSO was suggested to model a few parameters (tens) with fast forward routines. A number of theoretical studies refined both the mathematical and computational aspects of PSO, thus leading to a family of PSO algorithms (Ratnaweera et al. 2004; Ebbesen et al. 2012; Gou et al. 2017). Furthermore, due to the striking computational improvements of the last decade, PSO began to be applied to complex geophysical problems. Original works include 3-D gravity inversion (Pallero et al. 2015), microseismic event location (Lagos and Velis 2018), 2-D MT optimization (Pace et al. 2019a), full waveform inversion (Aleardi 2019) and joint inversion of multiple data sets (Paasche and Tronicke 2014).

The main applications of PSO to the geophysical inverse problem include the interpretation of:

- vertical electrical sounding (VES) (Fernández-Álvarez et al. 2006; Fernández Martínez et al. 2010a; Pekşen et al. 2014; Cheng et al. 2015; Pace et al. 2019b);
- gravity data (Yuan et al. 2009; Pallero et al. 2015, 2017, 2021; Darisma et al. 2017; Jamasb et al. 2019; Essa and Munschy 2019; Anderson et al. 2020; Essa and Géraud 2020; Essa et al. 2021);
- magnetic data (Liu et al. 2018; Essa and Elhussein 2018, 2020);
- multi-transient electromagnetic data (Olalekan and Di 2017);
- time-domain EM data (Cheng et al. 2015, 2019; Santilano et al. 2018; Pace et al. 2019c; Li et al. 2019; Amato et al. 2021);
- MT data (Shaw and Srivastava 2007; Pace et al. 2017, 2019a, c; Godio and Santilano 2018; Santilano et al. 2018) and radio-MT data (Karcıoğlu and Gürer 2019);
- self-potential data (Santos 2010; Pekşen et al. 2011; Göktürkler and Balkaya 2012; Essa 2019, 2020) and induced polarization (Vinciguerra et al. 2019);
- Rayleigh wave dispersion curve (Song et al. 2012) and full waveform inversion (Aleardi 2019).

### Objectives of the Review

The goal of this work is to review the recent advances in the application of PSO to different geophysical methods, e.g., electric, electro-magnetic, magnetic and gravity data. Our aim is to indicate the fundamental steps of the evolution of PSO methodologies that have been adopted to model the Earth’s subsurface and to undertake a critical evaluation of their benefits and limitations.

We investigate the state of the art of scientific literature regarding geophysical applications of the PSO algorithm. We illustrate some case studies of pivotal importance in electromagnetic, gravity, magnetic, self-potential, electric and seismic data. From an accurate literature review, we highlight the main contributions which have deployed PSO to solve some geophysical issues, to provide a new approach for geophysical modeling and to improve data interpretation and solution evaluation. Significant innovations have dealt with the choice of the geophysical measurement, the dimensionality of the problem to be solved (1-D, 2-D or 3-D), the parametrization of the geophysical model, the error associated to the data, the choice of the most appropriate PSO input arguments and the adoption of new releases of the algorithm to improve the model solution. We analyze the most-commonly adopted minimization functions, the flexibility of PSO if lateral and spatial constraints are imposed or if a priori information is introduced. We point out the possible limitations of existing works that could be improved by means of sophisticated variants of the PSO algorithm or by implementing a parallelized PSO to be run on clusters (e.g., cloud computing, high-performance-computing). These aspects could improve some of the existing simple applications of PSO to low-dimensional geophysical problems (tens of unknown parameters).

This work also focuses on a powerful application of PSO to solve multi-objective problems, such as joint optimization of multiple geophysical data sets. Multi-objective PSO has proven to be a valid method for an integrated interpretation of different geophysical measurements that present different resolutions, sensitivities, depths of investigation and/or error levels. The computational load of the PSO algorithm is much higher than that of the deterministic approach. However, it can be seen as a minor drawback if the algorithm arguments are correctly chosen and the code is parallelized. Finally, the work provides practical PSO guidelines to pave the way for further advances in this field.

## Particle Swarm Optimization: State of the Art

The original idea of the PSO algorithm was born from the observation of the choreography of bird flocks and schools of fish (Kennedy and Eberhart 1995). The way they share knowledge to search for food or find the best reciprocal distance in motion fascinated Kennedy and Eberhart (1995) so strongly that they proposed applying this evolutionary approach to the optimization of nonlinear problems. Pivotal references for computational swarm intelligence are Kennedy et al. (2001) and Engelbrecht (2007), that reports: “*PSO is a population-based search procedure where the individuals, referred to as particles, are grouped into a swarm. Each particle in the swarm represents a candidate solution to the optimization problem. In a PSO system, each particle is “flown” through the multidimensional search space, adjusting its position in search space according to its own experience and that of neighboring particles*”*.* Simple interactions between individuals yield a complex collective behavior, meaning that each individual is able to adapt and derive new and coherent behavior in case of changes in the external environment. The most striking feature of PSO is that every particle has a memory component that rules its behavior. “*The effect is that particles “fly” toward an optimum, while still searching a wide area around the current best solution. The performance of each particle (i.e. the “closeness” of a particle to the global minimum) is measured according to a predefined fitness function which is related to the problem being solved*”*.* The fitness function is the objective function of the optimization problem.

During the past two decades, PSO has been widely applied to solve optimization problems. The solution m is found after that the objective function, that is, the quantity to be optimized, is minimized (or maximized, depending on the problem) obeying or not some constraints. Fundamentals of the optimization theory can be found in Engelbrecht (2007). The typical properties of the most common (and challenging) optimization problems are

- Multivariate: there is more than one unknown
- Nonlinear: the objective function is non linear
- Constrained: the search space of the candidate solutions is restricted to specific regions according to equality or inequality constraints
- Multimodal or multi-solution: there is not only one clear solution, but a set of feasible candidate solutions referred to as local or global optima (whose mathematical definition is here omitted).

Another distinction of the optimization problems is between single-objective or multi-objective problems, meaning that there is one or more than one objective function(s) to be simultaneously optimized. In geophysics, an example of multi-objective optimization problem is the joint inversion of multiple geophysical data sets. Multi-objective PSO (MOPSO) of geophysical data is presented in Sect. 5.

### Classical PSO

A swarm is usually thought as a disorganized cluster of elements (insects, birds, fish, bacteria) apparently moving chaotically and following random directions. They are actually sharing their knowledge to pursue the goal of escaping from predators or keeping the best reciprocal distance in motion or searching for food. Social behavior allows particles to reach a specific objective and adapt to the environment. Therefore, the elements of the swarm can be regarded as massless and volume-less mathematical abstractions aiming at optimizing the objective function.

Assuming a nonlinear optimization problem affected by the non-uniqueness of the solution, as the geophysical inverse problem is, the set of the possible solutions can be imagined as a set of particles grouping in a swarm. The particles populate the search space of the problem solutions and change their position to fulfill the common objective. At the beginning of the optimization, the particles are initialized by being given uniformly distributed random position and null velocity. Then, the iterative swarming behavior begins. Each iteration, each particle is stochastically accelerated, on the one hand, toward its previous best position (i.e., where it minimized the objective function) and, on the other hand, toward the neighborhood best position (i.e., where any other particle minimized the objective function). These two basic approaches are referred to as exploration and exploitation, respectively. They compete in searching for the global minimum. While the exploration is associated to the cognitive behavior, that is, the memory component of the particle, the exploitation is related to the social behavior, that is, the convergence toward the leader.

The ruling equations of the standard PSO algorithm are

1 $${\varvec{v}}_{i}^{k + 1} = \omega^{k} {\varvec{v}}_{i}^{k} + \alpha_{1} \gamma_{1,i} \left( {{\varvec{P}}_{i} - {\varvec{x}}_{i}^{k} } \right) + \alpha_{2} \gamma_{2,i} \left( {{\varvec{G}} - {\varvec{x}}_{i}^{k} } \right)$$

2 $${\varvec{x}}_{i}^{k + 1} = {\varvec{x}}_{i}^{k} + {\varvec{v}}_{i}^{k + 1}$$

where ***x*** is the vector of the particle’s position composed of as many components as the problem unknowns, ***v*** is the velocity vector, *i* = [1, …, *N*], *N* is the number of particles forming the swarm, *k* is the current iteration number, ${\varvec{x}}_{i}^{k}$ and ${\varvec{v}}_{i}^{k}$ are the current vectors of position and velocity of the *i*th particle, respectively, *ω*^*k*^ is the inertia weight that balance the momentum remembered from the previous iteration, *α*_1_ is the cognitive acceleration towards the best particle position ***P***, also called “local best”, *α*_2_ is the social acceleration towards the best global position ***G*** (or “global best”) found by the group leader and *γ*_1_ and *γ*_2_ ∈ [0,1] are uniformly distributed random values which provide stochastic perturbation. The inertia weight *ω*^*k*^ usually linearly decreases from 0.9 (first iteration) to 0.4 (last iteration) (Shi and Eberhart 1998). However, many geophysical studies adopting PSO make use of constant inertia (*ω*) in the range 0.5–1, always less than 1.

**Fig. 3:**
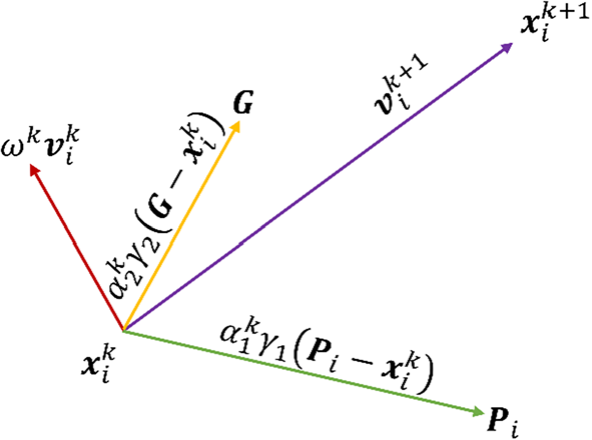
Graphical representation of the ruling equations of the PSO algorithm. (Adapted from Ebbesen et al. 2012)

At the beginning of the optimization (*k* = 0), the velocity vector $\left( {{\varvec{v}}_{i}^{0} } \right)$ is zero and the position vector $({\varvec{x}}_{{\text{i}}}^{0}$) is randomly initialized. Then (*k* > 0), the particle velocity $({\varvec{v}}_{i}^{k} )$ changes according to three terms: inertia component *ω*^*k*^, cognitive memory *α*_1_ and social attraction *α*_2_. Finally, the particle position ${\varvec{x}}_{i}^{k}$ is updated. Figure 3 clearly represents the graphical meaning of Eqs. 1 and 2. The *i*th particle moves from the position at iteration *k* (${\varvec{x}}_{i}^{k}$) to the following position (${\varvec{x}}_{i}^{k + 1}$) (purple arrow) as the resulting contribution of the three terms of Eq. 1: inertia *ω*^*k*^ (red arrow), cognitive attraction *α*_1_ (green arrow) and social attraction *α*_2_ (yellow arrow).

The PSO algorithm complies with the following three steps:

1. To evaluate the objective function for each particle
2. To update the individual and global best positions (**P** and **G**)
3. To update the velocity and position of each particle.

The previous steps are repeated as long as a valid ending condition is satisfied. The most common stopping criterion is to fix a maximum number of iterations. However, since the number of iterations is problem-dependent, there are some other stopping criteria to ensure an effective optimization of the objective function: an acceptable solution found, no improvements over a number of iterations, a normalized swarm radius tending towards zero or the objective-function slope tending towards zero (Engelbrecht 2007).

The values of the accelerations (*α*_1_ and *α*_2_) influence the way the particles explore the model space and change their trajectory with respect to the local and global bests. The accelerations values must obey the stability solution conditions (Perez and Behdinan 2007):

3 $$\alpha_{1} + \alpha_{2} < 4$$

4 $$\frac{{\alpha_{1} + \alpha_{2} }}{2} - 1 < \omega < 1$$

Equations 3 and 4 are derived from applying the necessary and sufficient condition for stability to the eigenvalues of the PSO equations written in matrix form. A generic PSO algorithm can be easily implemented thanks to practical guidelines both in MATLAB (Ebbesen et al. 2012) and in Python (Miranda 2018).

### PSO Variants and Input Arguments

A large number of different PSO variants have been developed since the first algorithm of Kennedy and Eberhart (1995) appeared. The early improvements of the code concentrated on the inertia weight *ω*^*k*^ (Shi and Eberhart 1998) and the acceleration coefficients *α*_1_ and *α*_2_ (Perez and Behdinan 2007) in order to improve both convergence speed and solution stability. The improvements regarding the inertia weight have been adopted by the majority of geophysical applications (Fernández Martínez et al. 2010a; Godio and Santilano 2018; Santilano et al. 2018). Standard PSO usually adopts constant values for the accelerations independently of the iterations.

Further developments of the standard PSO proposed some sophisticated adjustments to accelerate convergence and avoid the solution getting trapped in a local minimum. The most important PSO variants are: the fuzzy-adaptive PSO with fuzzy system tuning the inertia weight (Shi and Eberhart 1998), the self-organizing hierarchical PSO with time-varying acceleration coefficients (Ratnaweera et al. 2004), the hybrid quadratic PSO (Ying et al. 2006), the adaptive PSO (Zhan et al. 2009) and the individual-difference evolution PSO (Gou et al. 2017).

**Fig. 4:**
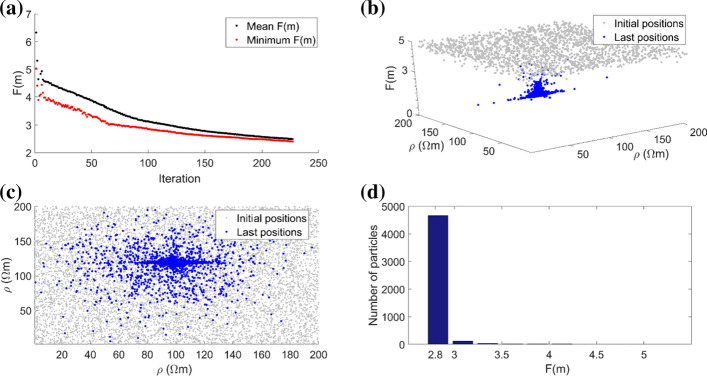
Objective function (*F*(***m***)) and particle positions at the end of PSO using $\alpha_{1}^{\max } = \alpha_{2}^{\max } = 2.75$ and $\alpha_{1}^{\min } = \alpha_{2}^{\min } = 0.5$: **a** calculated *F*(***m***) for the best particle (red dots) and the rest of the swarm (black dots) at each PSO iteration; **b** calculated *F*(***m***) as a function of the particle positions (*ρ*) in the search space, at the first (gray dots) and final (blue dots) iterations; **c** plain view of **b**; **d** final distribution of *F*(***m***) for the swarm at last iteration

**Fig. 5:**
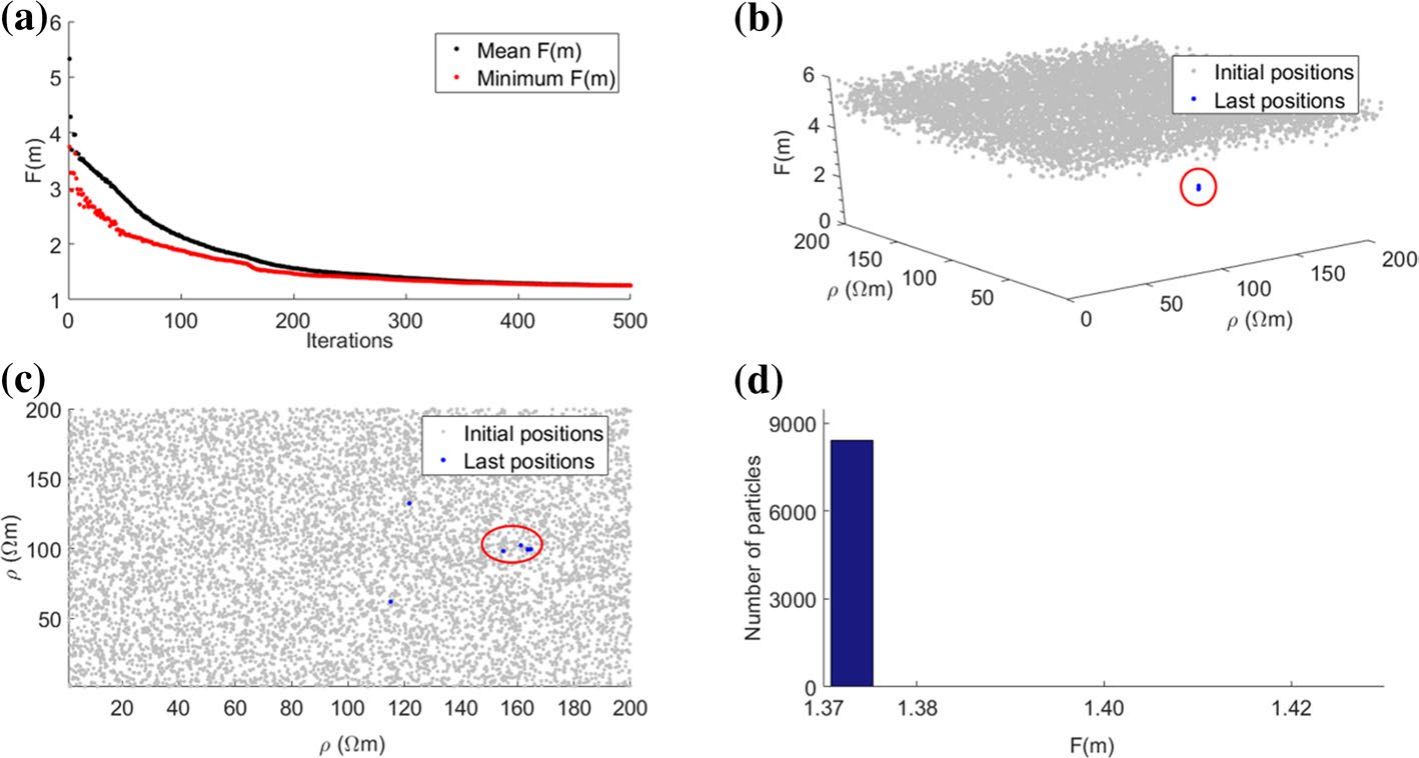
Objective function (*F*(***m***)) and particle positions at the end of PSO using $\alpha_{1}^{\max } = \alpha_{2}^{\max } = 2$ and $\alpha_{1}^{\min } = \alpha_{2}^{\min } = 0.5$: **a** Calculated *F*(***m***) for the best particle (red dots) and the rest of the swarm (black dots) at each PSO iteration; **b** Calculated *F*(***m***) as a function of the particle positions (*ρ*) in the search space, at the first (gray dots) and final (red circled blue dots) iterations; **c** plain view of **b**; **d** final distribution of *F*(***m***) for the swarm at last iteration

As introduced in the previous section, the main input arguments or tuning parameters of the PSO algorithm are:

- *The acceleration coefficients α*_*1*_* and α*_*2*_ The simplest variants of PSO adopt constant acceleration coefficients, that are usually equal to 2 since they are the benchmark values. Inspection of literature values reveals a wide range of accelerations setups if they are tuned to a specific geophysical case study. For this reason, cognitive acceleration is set in the range from 0.5 to 2 to 3.2. Social acceleration ranges instead from 1.1 to 2. A significant improvement in PSO efficiency has been observed by allowing the accelerations to vary with the iterations, as examined in Ratnaweera et al. (2004) with a thorough sensitivity analysis. This variant is called hierarchical PSO with time-varying acceleration coefficients (HPSO-TVAC) and takes the social and cognitive behavior of particles into account to enhance the solution convergence and stability. Useful examples about the influence of the PSO accelerations on the geophysical models can be found in Fernández Martínez et al. (2010a; b) and Pace et al. (2019a). The ruling equations of the HPSO-TVAC are slightly different from Eqs. 1 and 2 in that the acceleration parameters are k-dependent:
  5 $${\varvec{v}}_{i}^{k + 1} = \omega^{k} {\varvec{v}}_{i}^{k} + \alpha_{1}^{k} \gamma_{1} \left( {{\varvec{P}}_{i} - {\varvec{x}}_{i}^{k} } \right) + \alpha_{2}^{k} \gamma_{2} \left( {{\varvec{G}} - {\varvec{x}}_{i}^{k} } \right)$$
  6 $${\varvec{x}}_{i}^{k + 1} = {\varvec{x}}_{i}^{k} + {\varvec{v}}_{i}^{k + 1}$$
  where the equation terms have already been defined. The HPSO-TVAC approach states that, at the beginning of the optimization, *α*_1_ is larger than *α*_2_ and then they linearly reverse. In this way, at the start, the diversity of the swarm ensures the search space exploration (high *α*_1_^*k*^), and, at the end, the exploitation of the best regions and the convergence towards the global minimum are enabled (high *α*_2_^*k*^). The resulting adaptive behavior is hence enhanced. In more detail, the cognitive and social accelerations obey Eqs. 3 and 4 and change according to:
  7 $$\alpha_{1}^{k} = \alpha_{1}^{\max } - (\alpha_{1}^{\max } - \alpha_{1}^{\min } )\left( {\frac{k - 1}{{\max \left( k \right) - 1}}} \right)$$
  8 $$\alpha_{2}^{k} = \alpha_{2}^{\min } + (\alpha_{2}^{\max } - \alpha_{2}^{\min } )\left( {\frac{k - 1}{{\max \left( k \right) - 1}}} \right)$$
  where *α*^*k*^ is the acceleration value at iteration *k*; *α*_1_^max^ and *α*_2_^max^ are the maximum values for the cognitive and social accelerations, respectively; *α*_1_^min^ and *α*_2_^min^ are the minimum values for the cognitive and social accelerations, respectively; and max(*k*) is the maximum number of iterations set for the optimization (Engelbrecht 2007 and references therein). Therefore, at the first iteration (*k* = 1), $\alpha_{1}^{k = 1} = \alpha_{1}^{{{\text{max}}}}$ and $\alpha_{2}^{k = 1} = \alpha_{2}^{\min }$, while, at the last iteration (*k* = max(*k*)), $\alpha_{1}^{{k = {\text{max}}\left( k \right)}} = \alpha_{1}^{{{\text{min}}}}$ and $\alpha_{2}^{k = \max \left( k \right)} = \alpha_{2}^{\max }$. The best range of the acceleration values ensuring the convergence and stability of the solution has been tested and identified for several benchmark functions (Ratnaweera et al. 2004; Fernández Martínez et al. 2010a, b). Starting from their results, and obeying Eqs. 3, 4, 7 and 8, Pace et al. (2019a) performed some tests to assess the influence of several acceleration values on the solution of the 2-D MT inverse problem. That sensitivity analysis outlined $\alpha_{1}^{{{\text{max}}}} = 2$, $\alpha_{1}^{{{\text{min}}}}$ = 0.5, $\alpha_{2}^{\min } = 0.5$, and $\alpha_{2}^{\max } = 2$ as optimal acceleration values for a robust minimization of the objective function (see Fig. 5 compared to Fig. 4). These accelerations were also adopted for PSO of TDEM and VES data (Pace et al. 2019b; Amato et al. 2021).
- *The stopping criterion/criteria adopted to end the iterations* The PSO algorithm is iterated enough to guarantee as far as possible minimization of the objective function. Many PSO applications have commonly adopted the maximum number of iterations as the unique stopping criterion (Fernández Martínez et al. 2010b; Pallero et al. 2017; Godio and Santilano 2018; Santilano et al. 2018). However, the number of iterations is problem dependent and its arbitrary choice can lead to either an ending before the solution convergence or unnecessary computation (Engelbrecht 2007). Recent studies took into account the trend of the objective function during the minimization (Pace et al. 2019a, b). PSO ran as long as the objective function did improve for a significant number of consecutive iterations (e.g., 100) or, if this condition was not satisfied, up to a maximum number of iterations (e.g., 2000). Another stopping criterion useful in geophysical modeling is the minimum root-mean-square error (RMSE) equal to 1 (± 10% of tolerance), to avoid the fitting of the data below their uncertainty (deGroot‐Hedlin and Constable 1990). Depending on the application, multiple stopping criteria can be combined. Additional stopping criteria consider the performance of the optimization, such as the slope of the objective function, the distribution of the particles, the standard deviation of the particle positions (Kennedy et al. 2001).
- *The swarm size N, i.e., the number of particles forming the swarm* N influences the way the particles distribute over the search space to guarantee the exploration of the possible solutions. The swarm size must be sufficiently high to ensure a wide initial coverage of the search space, so that the particles can efficiently explore all of the regions potentially hosting the global minimum. This behavior is missed if the swarm is too small, although giving the advantage of unburdening the computational complexity. An interesting analysis on the relation between the swarm size and the computational complexity can be found in van den Bergh and Engelbrecht (2001). The number of particles is a problem-dependent parameter and it is usually set proportional to the number of unknowns, that is, in geophysical modeling, the number of the model parameters. The ratio between the problem unknowns and the number of particles was suggested to be between 8 and 12 times the unknowns by Engelbrecht (2007, p. 241) for GA and by Fernández Martínez et al. (2010a) for PSO. The sensitivity analysis performed in Pace et al. (2019a) considering a 2-D MT synthetic model outlined the multiple of 9 as the best ratio, giving the preferred inversion model, the minimum number of iterations and the second shortest runtime.
- *The initialization settings* The initialization of the optimization is another essential feature of PSO. At the beginning, the particles in the search space are by default randomly distributed and bounded between a minimum and maximum value. This range is constant during the optimization but can vary from each layer (or group of layers or cells) to another (Godio and Santilano 2018; Pallero et al. 2021). The decision of the lower and upper boundaries is problem dependent and should be coherent with the desired coverage of the search space of solutions. Tight boundaries can decrease the convergence time, but must be chosen with caution as long as there is a reasonable confidence in the expected solution that avoids any bias. In addition, the adoption of tight boundaries can represent a way to add reliable a priori information to the optimization (e.g., from wells), even though it represents a strong constraint. After the random initialization, the adaptive behavior controls the position updating and a stochastic perturbation is guaranteed by *γ*_1_ and *γ*_2_ (Eqs. 1 or 5). Derivative-based inversion algorithms usually deploy a homogeneous or a priori model as starting model to initialize the geophysical inversion. The a priori information is derived from geologic (well-log) data or other geophysical methods. The key factor of global search algorithms such as PSO is that they are independent of the starting model and, consequently, do not necessarily require external information to initialize the optimization (Pace et al. 2019b). However, it may also be possible to use a priori information to partially influence the swarm behavior by setting the initial position (i.e., a proposed model) for a small portion of the swarm (e.g. 5% of the particles). In this way, if the data agree with a priori information, the optimization is influenced, otherwise the optimization disregards the information and searches for a valid solution thanks to its adaptive behavior (Pace et al. 2019a).

The choice of the input arguments influences the optimization performance and hence the final solution of the problem investigated. Given the same case study of PSO applied to 2-D MT synthetic data, Figs. 4 and 5 show an example of poor and successful convergence, respectively (Pace et al. 2019a). The two outcomes result from different acceleration coefficients, that is, $\alpha_{1}^{\max } = \alpha_{2}^{\max } = 2.75$ and $\alpha_{1}^{\min } = \alpha_{2}^{\min } = 0.5$ in Fig. 4 and $\alpha_{1}^{\max } = \alpha_{2}^{\max } = 2$ and $\alpha_{1}^{\min } = \alpha_{2}^{\min } = 0.5$ in Fig. 5. Figure 4 shows that the minimization of the objective function was not effective. Figure 4a plots the objective function calculated for the best particle (red dots) and the mean value of the remaining particles (black dots) as a function of the iterations. Figure 4b plots the objective function values of the swarm as a function of the search space, i.e., two cells of the 2-D grid, at the first (gray dots) and final (blue dots) iterations. Figure 4c is the plain view of Fig. 4b and demonstrates that the distribution of the particles in the search space was still scattered at the last iteration. Figure 4d is the histogram of the objective function calculated for all the particles at the last iteration and reveals that the optimization did not end in a convergence state because the minimum value of the objective function was not reached by the totality of the particles. Figure 5 shows that PSO ended in true convergence because the particles converged toward a single position (the blue dots in Fig. 5b, c) and toward the same value of objective function as demonstrated by the histogram of Fig. 5d. Figures 4 and 5 confirm that the choice of the optimal input arguments ensures a robust minimization and convergence.

**Fig. 6:**
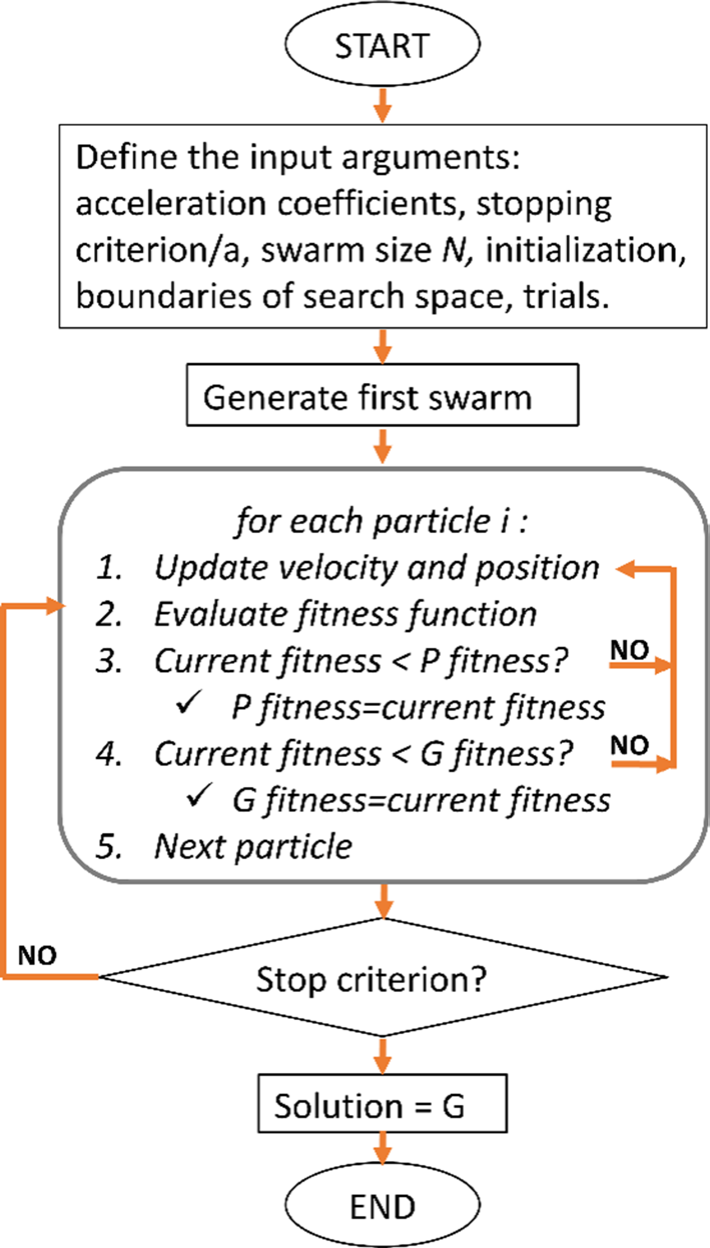
The PSO algorithm flowchart. ***P*** is the local best solution and ***G*** is the global best solution (from Pace et al. 2019a)

The PSO flow chart is shown in Fig. 6. This procedure can be repeated several times (or “trials”) to assess the variability on the solution resulting from the random initialization. In fact, the final solutions coming from different initial random distributions are quite similar but not identical, as shown in Santilano et al. (2018) for 1-D MT. The solution with the lowest RMSE is usually selected as the preferred optimized (inversion) model. An associated criterion to select the preferred solution from a set equivalent solutions obtained from the PSO trials is to show both the solution with the minimum RMSE and a subset of solutions within the 5–15% of the minimum RMSE (Pallero et al. 2017; Godio et al. 2020; Amato et al. 2021). This criterion has the advantage of showing the equivalence region within a tolerance of the RMSE. Moreover, the swarm can be inspected at the last iteration by calculating the mean (or median) and standard deviation of each model parameter to estimate a model solution based on these statistical quantities (Fernández Martínez et al. 2010b; Godio and Santilano 2018; Pallero et al. 2018, 2021). Finally, to assess the uncertainty of the final outcome, it is recommended to analyze the a posteriori probability density (*ppd*) function of each model parameter. Different approaches were explored considering the *ppd* of the swarm either for a single PSO run or for several PSO trials (several runs with equal settings) (Santilano et al. 2018; Pallero et al. 2018, 2021). It may happen that the most occurred value of a parameter in the *ppd* curve is different from the value it assumed in the lowest-RMSE model. This inspection definitely overcomes the limitation of the unique solution found by local search methods.

### Multi-objective PSO

PSO can be adopted to solve multi-objective problems as a tool for joint optimization of multiple geophysical data sets. Theory about MOPSO and its variants is given in Coello et al. (2004, 2007), Reyes-Sierra and Coello Coello (2006) and Tripathi et al. (2007).

Joint inversion of multiple data sets can significantly improve their modeling by overcoming the intrinsic limitations of each geophysical method. The advantages in combining different geophysical measurements using a single inversion scheme have been clear since the first introduction of joint inversion methods (Vozoff and Jupp 1975; Yang and Tong 1988) and recent advances (Hering et al. 1995; Musil et al. 2003; Gallardo and Meju 2003; Moorkamp et al. 2011; Sen and Stoffa 2013). Joint inversion is though affected by data compatibility because field data of different geophysical methods usually present different depths of investigation, resolutions, sensitivities and/or error levels. Consequently, joint inversion may provide as a result a variety of conflicting models or biased results. The choice of a proper weighting factor between the objective functions is critical but may resolve the conflict (Commer and Newman 2009; Akca et al. 2014; Meqbel and Ritter 2015).

Multi-objective evolutionary algorithms (MOEAs) are deployed for joint inversion of multiple data with the advantage of avoiding the simplification of a multi-objective problem (joint inversion) into a succession of single-objective optimization problems adopting user-dependent weighted objective functions. Another advantage of performing joint inversion with MOEAs is that these global search methods are not dependent on the first assumption of the starting model. The striking feature of performing joint optimization with MOEAs is that the objective function is a vector function with as many components as the different geophysical data sets to be optimized.

**Fig. 7:**
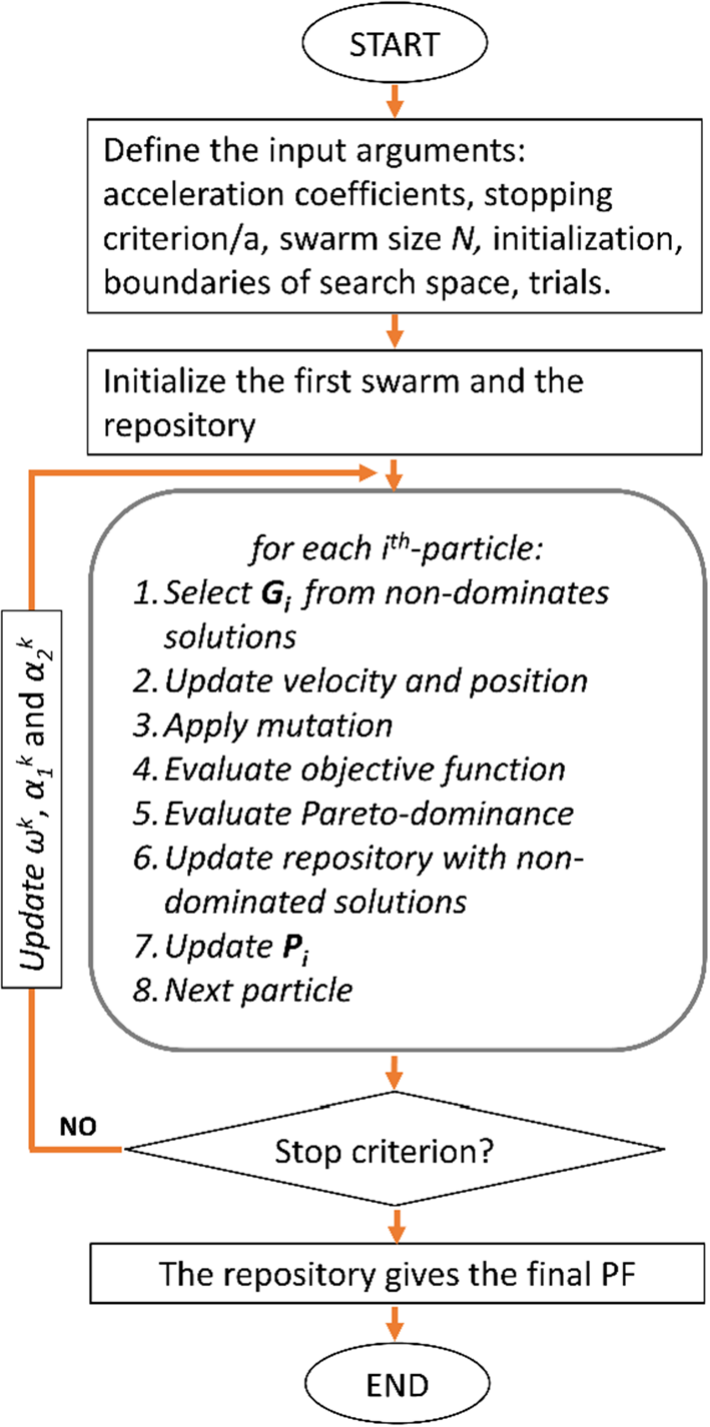
The MOPSO-algorithm flowchart. ( Adapted from Pace et al. 2019b)

The common approach is to exploit the economic concept of Pareto optimality that avoids the ranking of the vector components (Edgeworth 1881; Pareto 1896; Baumgartner et al. 2004). This principle identifies a range of compromises as feasible solutions of the geophysical problem. It states that a candidate solution is considered *Pareto-optimal* if there is not another candidate solution that minimizes one objective without degrading the other objective. The set of solutions obeying this criterion are called non-dominated solutions and form the *Pareto-optimal set*. Its projection onto a surface creates the so-called *Pareto Front *(*PF*), which is a tradeoff surface showing which component of the objective function is mostly minimized. The *PF* is also useful to infer the data compatibility (Dal Moro and Pipan 2007; Schnaidt et al. 2018; Pace et al. 2019b). The ruling equations of MOPSO are the same of PSO (Eqs. 1 and 2, or 5 and 6) with the only difference that in single-objective PSO the leader ***G ***is the best particle of the swarm, while in MOPSO the leadership belongs to the non-dominated solutions. The non-dominated solutions are stored in a *repository*, from which ***G***_***k***_ is selected at each iteration according to a quasi-random criterion based on the most crowded regions of the objective space. The flowchart of the MOPSO algorithm is schematically shown in Fig. 7.

MOPSO has proven to be a valid method for an integrated interpretation of different geophysical measurements presenting different resolutions, sensitivities, depths of investigation and/or error levels. Significant examples of MOPSO are joint optimization of electric and electromagnetic data (Cheng et al. 2015; Pace et al. 2018, 2019b), ground-penetrating-radar (GPR) and P-wave seismic travel times (Tronicke et al. 2011; Paasche and Tronicke 2014). Some of these case studies are reviewed in Sect. 5. For the sake of completeness, another MOEA adopted in geophysics is the Nondominated Sorting GA (NSGA-III) (Deb and Jain 2014). NSGA has actually been more explored than MOPSO and applied to the inversion of: Raleigh-wave dispersion curves and reflection travel times (Dal Moro and Pipan 2007), surface wave dispersion and horizontal-to-vertical spectral ratio (Dal Moro 2010), seismic and well-log data for reservoir modeling (Emami Niri and Lumley 2015), magnetic resonance and VES data (Akca et al. 2014), AMT and broad-band MT data (Schnaidt et al. 2018), and receiver functions, surface wave dispersion and MT data (Moorkamp et al. 2011).

### Minimization of the Objective Function Using PSO

The interpretation of geophysical data is accomplished by solving the inverse problem, which provides a model of the distribution in the Earth’s subsurface of the physical parameters investigated. The inversion consists in finding the model parameters ***m***, considering the observed data ***d***_obs_, and applying the functional *F*, which entails the physics of the geophysical technique investigated.

9 $$F\left( {\varvec{m}} \right) = {\varvec{d}}_{{{\text{obs}}}}$$

The basic concept of PSO application to geophysics is that each particle of the swarm represents a possible solution of the inverse problem. Since this solution is affected by non-uniqueness, the search space of solutions needs to be fully explored to find the best model, which fits the observed data. This need is fulfilled by the adaptive and swarming behavior of the particles. Differently from derivative-based inversion techniques, solving the inverse problem by means of PSO means that the forward functional *F* calculates the predicted responses for each assumed model ***m***. The calculated responses are compared with the observed data for each particle. The final goal is the minimization of the objective function. The particle with the lowest objective function value is awarded with the global best position ***G*** and exerts influence on the neighbors depending on the social acceleration *α*_2_ (Eq. 1). At the end of the swarming, the optimized solution is expected to be identified.

The objective function to be minimized is a misfit function that computes the differences between the observed data and the predicted responses calculated by the forward modeling from an assumed Earth model ***m***. The objective function can also include a further term that manages the model roughness according to the concept of Occam’s inversion (Constable et al. 1987). It is known that in derivative-based inversion techniques, the closest fitting between observed and predicted data brings to the maximum roughness, i.e., spurious structures. A smooth model avoids the over-interpretation of the data beyond their resolving capability. The concept of “Occam-like regularization” has also been applied to PSO in order to minimize not only the data deviations but also the roughness of the model ***m*** (Godio and Santilano 2018; Santilano et al. 2018; Pace et al. 2019a, b).

The general objective function to be minimized is usually defined as the norm of the misfit between the observed data and calculated responses. The Euclidean or *L*_2_ norm is commonly adopted, but the geometric, harmonic and *L*_1_ norms can also be found in the literature for particular geophysical applications (see examples in Sen and Stoffa 2013). An additional term can be potentially included to regulate the model roughness:

10 $$F\left( {\varvec{m}} \right) = \frac{{\phi_{{\text{o}}} - \phi_{{\text{c}}} }}{{{\varvec{\sigma}}_{\phi } }} + \lambda \parallel \log_{10} \left( {\partial {\varvec{m}}} \right)\parallel$$

where *ϕ*_o_ is the observed signal; *ϕ*_c_ is the calculated response; the difference in $\left\| \cdot \right\|$ is normalized by the corresponding errors (*σ*_*ϕ*_) on the observed data; *λ* is called the Lagrange-multiplier, or smoothing parameter. The right-hand side of Eq. 10 is composed of two terms: the first one assesses the distance of the observed data from the responses calculated by the forward modeling; the second term is non-obligatory and addresses the minimization of the roughness of the model, by using the smoothing parameter *λ* on the first derivative of the model ***m***. The proper value of *λ* is usually chosen following the L-curve criterion, which identifies the optimal tradeoff between the minimum data misfit achievable and the minimum model norm, i.e., unnecessary structure (or roughness) of the final model (Farquharson and Oldenburg 2004). A high value of *λ* results in a smooth model penalizing the misfit, while, on the contrary, a low *λ* yields a minimum data misfit but sharp contrasts (roughness) between the layers (or cells) of the model.

As previously explained, the objective function of joint optimization has as many *j*th components as the data sets to be optimized.

11 $$F_{j} \left( {\varvec{m}} \right) = \frac{{\phi_{{\text{o}}} - \phi_{{\text{c}}} }}{{{\varvec{\sigma}}_{\phi } }} + \lambda_{j} \parallel \log_{10} \left( {\partial {\varvec{m}}} \right)\parallel$$

where *λ*_*j*_ addresses the different level of smoothing required by the particular geophysical data set.

The validity of the preferred PSO model is usually assessed by means of a posteriori analysis among the different solutions given by the several PSO trials. The equivalence region of the solution can be inspected by calculating the mean (or median) and the standard deviation among the final solutions. The uncertainty of the preferred model is evaluated with the *ppd* function of the parameter values at each layer (or cell). The shape of the *ppd* can feature a multimodal or a unimodal behavior. If the solutions are analyzed at the last iteration (or also, after several PSO trials with equal settings), and if the model parametrization is appropriate, a unimodal distribution is the indicator of solution convergence and good quality. A multimodal distribution instead reflects the complexity and ill-posedness of the inverse problem (Fernández Martínez et al. 2010b; Godio and Santilano 2018; Pallero et al. 2018). The a posteriori analysis of the final PSO solutions should become a crucial step in the discussion of PSO solutions.

## PSO of Electromagnetic Data

The solution of the electromagnetic (EM) inverse problem is a challenge for the scientific community due to the high nonlinearity and ill-posedness of the problem, according to the definition by Hadamard (1902).

In particular, the solution of the inversion of MT data is not unique and is in a certain way unstable. The application of derivative-based inversion schemes results in a high dependence of the solution on the starting model that initializes the deterministic inversion. Nowadays, deterministic algorithms are by far conventional for the inversion of MT data. The scientific community can indeed exploit the state of the art of well-established algorithms for 1-D, 2-D and 3-D inversion, such as 1-D Occam inversion (Constable et al. 1987) and the 2-D and 3-D nonlinear conjugate gradient (NLCG) schemes (Rodi and Mackie 2001; Kelbert et al. 2014).

The computational complexity of the EM forward problem drastically increases with its dimensionality. For this reason, the application of stochastic population-based algorithms is not conventional and represents a challenge that has recently been solved for 1-D and 2-D inversion but not yet addressed for 3-D inversion.

### 1-D MT

The earliest applications of PSO to the 1-D MT inverse problem were presented in conferences proceedings (Godio et al. 2016; Patel et al. 2016; Grandis and Maulana 2017) and published in full articles (Shaw and Srivastava 2007; Godio and Santilano 2018; Santilano et al. 2018). At the same time, research focused on the solution of the 1-D MT inverse problem by adopting the Markov Chain Monte Carlo (MCMC) method (Grandis et al. 1999; Mandolesi et al. 2018; Xiang et al. 2018; Conway et al. 2018) and the SA algorithm (Dosso and Oldenburg 1991).

Godio and Santilano (2018) proposed a comprehensive work to describe the adoption of the PSO in MT and to state the validity of PSO applied to the 1-D MT inverse problem. Even though 1-D MT inversion is well-documented, the study represents a benchmark for the application of SI to EM methods and to more complicated problems such as 2-D MT, joint inversion and other geophysical methods.

The study by Godio and Santilano (2018) firstly focuses on the optimization of the parameters of a blocky earth model, i.e., a few-layered model, which is a common practice for the use of global search methods in geophysical inversion. In the blocky optimization, the unknown vector is composed of the resistivity and thickness values of each layer and the search space is implemented accordingly. The PSO was able to solve the 1-D inverse problem and to find the optimum blocky model, even though the simplification to a blocky scheme can be seen as a limitation. An innovation is the implementation of the “Occam-like” scheme to obtain a smooth earth model with many layers. In the Occam-like scheme, the model vector is composed of the resistivity of each layer with fixed thickness (logarithmically increasing). In the objective function, the smoothing parameter (see Eq. 10) acts on a differential operator of the model parameters to minimize the model roughness. This is the first attempt to adapt the classical deterministic Occam inversion (from Constable et al. 1987) to a global optimization method.

The random initialization produces slightly different results if the PSO run is repeated for several “trials” with the same data and settings. Several trials of the same PSO setup generate a set of final models, that can be statistically analyzed with an “a posteriori” distribution to verify their quality. The large is the scattering of the results, the poorer is the quality of the solution, and vice versa. The a posteriori distribution can be affected by: the propagation of the data uncertainties, the limit of the forward modeling to reproduce the complexity of the true resistivity distribution and the conditioning of the optimization process.

**Fig. 8:**
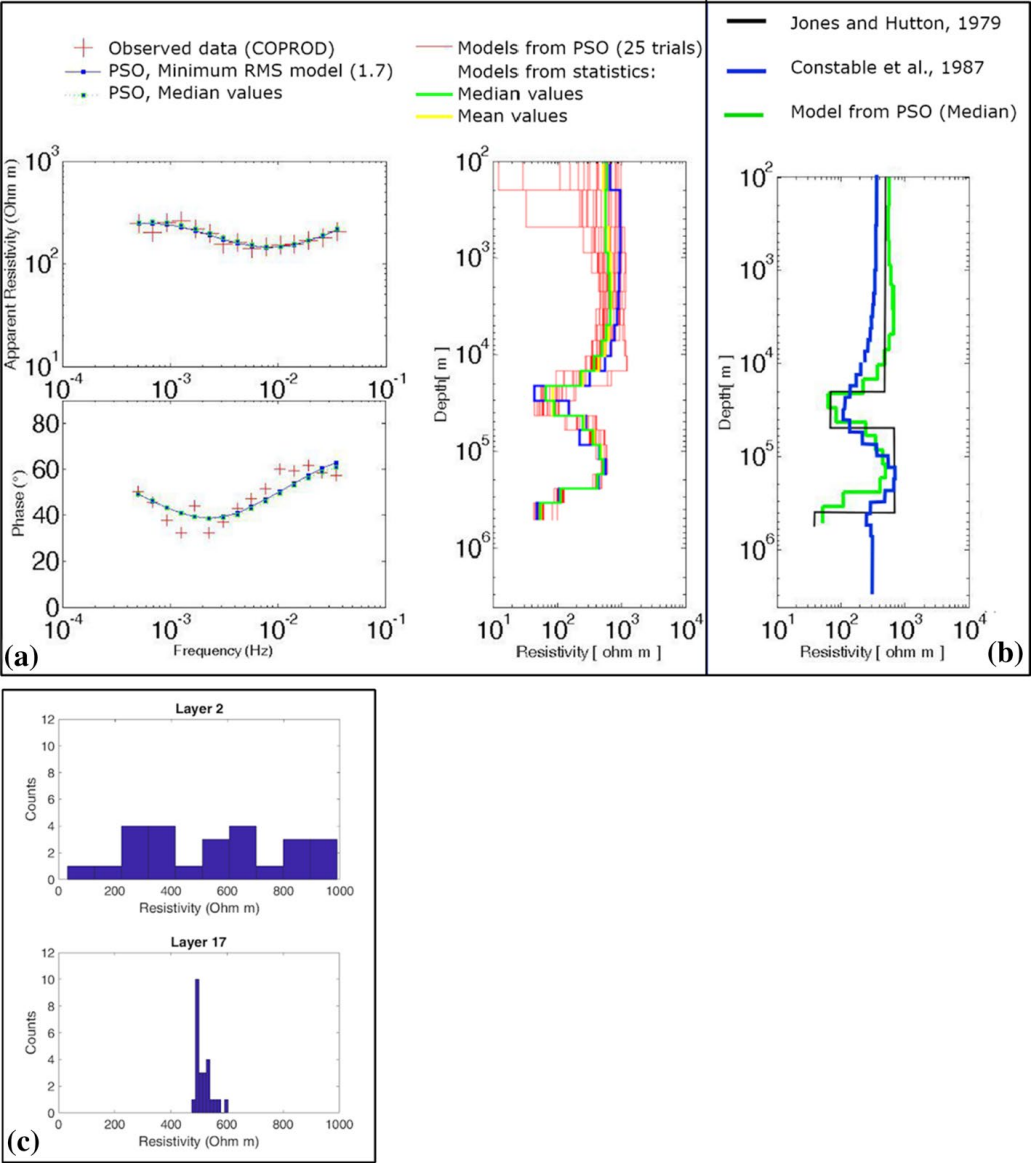
**a** PSO of COPROD MT data (modified from Godio and Santilano 2018 after Jones and Hutton 1979): on the left, data fitting for apparent resistivity and phase between observed data (red cross) and calculated response (blue dotted line) and on the right, the 1-D resistivity models from PSO (red lines), the mean model (yellow line) and the median model (green line); **b** comparison between the PSO model (green) and the models from Constable et al. (1987) (blue) and from Jones and Hutton (1979) (black); **c** the a posteriori probability density (*ppd*) function of layers 2 and 17 from the PSO trials in **a**: the histograms show a multimodal and a unimodal distribution, respectively

The authors apply PSO to real and synthetic cases from audio-MT and long period MT data. In particular, the COPROD dataset (Jones and Hutton 1979) was used because it is available to the scientific community for testing new algorithms (Fig. 8). In this case study the swarm is composed of 300 particles and the input arguments are constant: the inertia weight is equal to 0.9, the cognitive and social accelerations equal to 0.5 and 1.5, respectively. 25 trials are run, each for 200 iterations. The PSO, as it is conceived, provides the best solution that emerges thanks to the intelligent behavior of the particles.

### 2-D MT

The inversion of 2-D MT data is usually based on algorithms such as Occam, NLCG, Gauss–Newton (GN) and other variants, which are now widely recognized as milestones among 2-D and 3-D MT inversion codes (Siripunvaraporn 2012). These derivative-based inversion schemes ensure convergence in few iterations, but the final solution depends on the initial assumption of the starting model. If a homogeneous half-space is adopted as a starting model, some trials have to be done to define the most appropriate value of the electrical resistivity to start with, depending on the data set and inversion code (Miensopust et al. 2013; Tietze et al. 2015). Otherwise, the inversion should be initially constrained by an a priori model that can resolve the non-uniqueness of the solution by using information from well-log data (Yan et al. 2017a), seismic data (Yan et al. 2017b), MT data (Santilano 2017) or other geophysical methods. However, the initial guess can bias the model solution and its interpretation (Tietze et al. 2015).

To date, only a limited number of works have applied metaheuristic methods to the 2-D MT inverse problem. A preliminary application of PSO to 2-D MT and audio-MT data considered only synthetic data (Pace et al. 2017). PSO of 2-D MT data was performed to characterize a sedimentary basin (Pace et al. 2019a) and a geothermal system (Pace et al. 2019c, 2020), after an accurate validation of the method on two MT synthetic models of different complexity.

To properly address the complexity of the 2-D MT inverse problem, the efficiency of the PSO algorithm was improved by applying the PSO variant called HPSO-TVAC (Eqs. 5, 6). In fact, the assumption of constant values for the social and cognitive accelerations is not adequate for the 2-D inverse problem due to its high dimensionality and complex searching behavior. The sensitivity analysis on the time-varying accelerations improved the convergence speed of the algorithm and prevented the solution from being trapped in some local minima. In addition, a new parallelized version of PSO to be run on a high performance computing (HPC) cluster was effective in overcoming the time-consuming nature of PSO, which is computationally demanding, like the other global search algorithms. (The parallelization of the PSO tests performed on the HPC led to runtime savings of more than 80% if 24 cores were adopted instead of the 4 cores of a common laptop computer.)

The first PSO application to 2-D MT field data regards the COPROD2 data set, that is the benchmark to test new 2-D MT inversion methods and to compare numerous inversion solutions (Jones 1993a). The COPROD2 data set is a 400 km east–west profile crossing a 2-D geoelectrical structure in Saskatchewan and Manitoba, Canada (Jones and Savage 1986). The data set presents low impedance errors (< 2%) and includes the correction of the static shift. To perform PSO of COPROD2 data, a subset of 20 sites was selected to focus only on the center of the profile. Since the responses below 10 s present a one-dimensional behavior, the selected period range was from 10.67 to 910.2 s (Martí et al. 2009). The errors on the data were kept as original for both transverse electric (TE) and transverse magnetic (TM) apparent resistivity and phase. The 2-D model mesh was divided into 10 layers, from 1.8 to 60.5 km of depth, whose thickness increased logarithmically with depth. The 200-km-long mesh was divided into 34 bricks to include the boundary conditions. The total number of cells was 340. Since some structures of the region are known to be highly conductive, the lower boundary of the search space was 0.1 Ωm. The upper boundary of the upper layers (up to 5 km of depth) was 10 Ωm since the superficial sediments are known to be far more conductive than the resistive basement. The upper boundary of the underlying layers was 1000 Ωm.

As regard the PSO input arguments, the cognitive acceleration $\alpha_{1}$ linearly decreased from 2 to 0.5 and the social acceleration $\alpha_{2}$ linearly increased from 0.5 to 2. The population size was 2500 particles, proportional to the number of cells. In order to retrieve the optimal value of the Lagrange multiplier *λ* (Eq. 10), a sensitivity analysis was performed on five different values in the range between 0.001 and 10. Since the problem is 2-D, *λ*_*x*_ and *λ*_*z*_ were contextually analyzed with the same value and the optimal value was chosen as the point of maximum curvature in the plot of data misfit versus model norm. The best tradeoff value was equal to 0.1 for both *λ*_*x*_ and *λ*_*z*_. A priori information was not given: the optimization started with a completely random initialization.

**Fig. 9:**
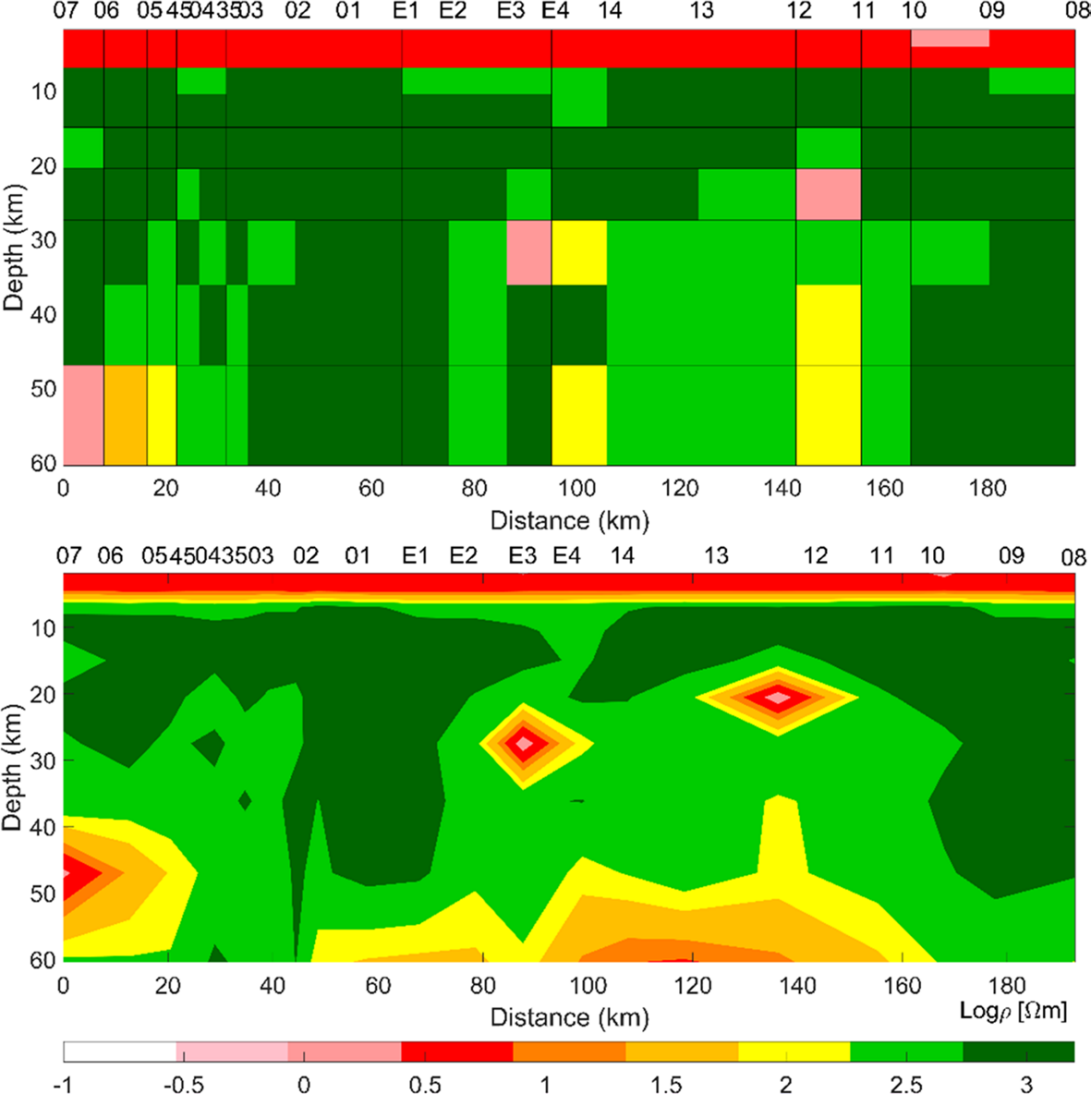
(Top) Resistivity model of COPROD2 MT data from PSO computation, after 6000 iterations. Lagrange multiplier *λ* = 0.1 (from Pace et al. 2019a); (bottom) interpolated model for interpretation

The final model from COPROD2 data was computed after 6000 iterations and is depicted in Fig. 9. The most significant feature of this model is the low-resistivity anomalies below the station E3–E4 and 12–11 at around 20 to 35 km of depth. The PSO output is well comparable with the models published in the literature since the low-resistivity anomalies were identified in the same regions (Jones 1993b).

**Fig. 10:**
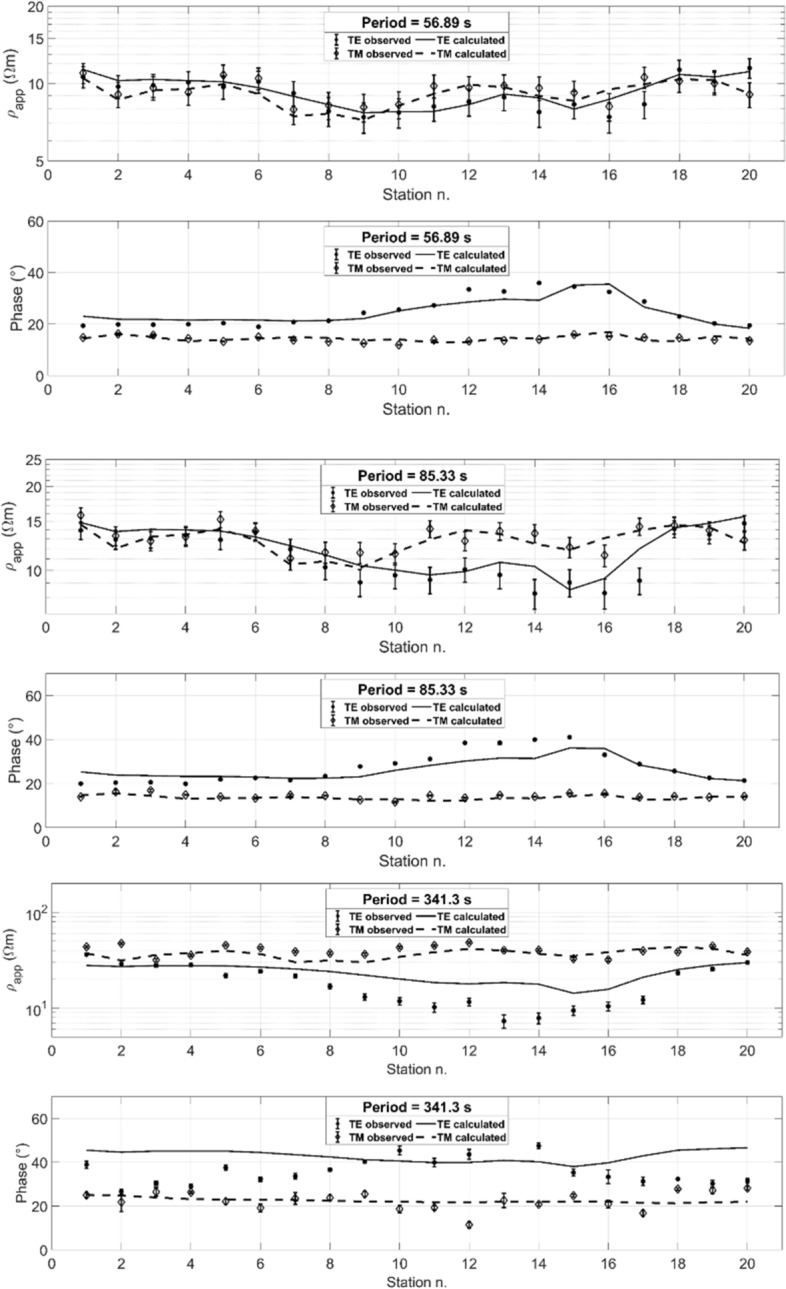
Fitting curves between observed apparent resistivity (*ρ*_app_) and phase, and predicted responses at selected periods: 56.9 s, 85.3 s, 341.3 s (from Pace et al. 2019a). Observed data include error bars and are marked with dots for TE and diamonds for TM. Calculated responses are plotted with solid line for TE and dashed line for TM. The optimization was randomly initialized

Figure 10 shows the observed data and predicted responses for the apparent resistivity (*ρ*_app_) and phase at selected periods for the 20 stations. The observed data are marked with dots for TE and diamonds for TM, and predicted responses are plotted with a solid line for TE and a dashed line for TM. The final RMSE was 2.42. PSO of COPROD2 data was executed on a 24-core node of an HPC cluster for academic research, with a total runtime of 8 h. (The CPU model of the single node was an Intel Xeon E5-2680 v3 2.50 GHz (turbo 3.3 GHz) with 128 GB of RAM.)

### PSO of MT and TDEM Data for Static Shift Correction

Chave et al. (2012) consider the distortion of regional electric fields by local structures as the greatest problem of the MT method. This effect, also known as “static shift”, is a galvanic distortion of MT data caused by near-surface small-scale heterogeneities or topography. The effect is a frequency-independent shift of the MT apparent resistivity curve by an unknown multiplier (constant on a logarithmic scale) that does not affect the MT phase (Jones 1988).

The adoption of TDEM (or TEM) data to solve the static shift of an MT sounding has been extensively studied in the literature (e.g., Sternberg et al. 1988; Pellerin and Hohmann 1990; Meju 1996). Among the several correction techniques, static-shift correction by means of TDEM data is quite common for geothermal exploration (e.g., Árnason 2015), being MT and TDEM widely used for imaging geothermal systems (Spichak and Manzella 2009; Muñoz 2014). The TDEM method is considered an effective means of correcting the static shift of distorted MT data because TDEM measurements are not (or slightly) affected, by this kind of distortion.

Santilano et al. (2018) discuss the application of the PSO algorithm to overcome the galvanic distortion by providing a quantitative estimate of the static shift. The authors propose a joint analysis of MT and TDEM data. The method is based on the correlation between the time-domain diffusion depth and the frequency-domain skin depth, and the computation of a TDEM apparent resistivity from the measured voltage (e.g., Spies and Eggers 1986). At a certain site and at the same depth of penetration, the TDEM time *t* (ms) is assumed to be equivalent to the MT period *T* (s) according to the equality proposed by Sternberg et al. (1988):

12 $$t = 194 \cdot T$$

Therefore, the TDEM response is converted into an equivalent MT period. Obviously, the overlapping of the two apparent resistivity curves (TDEM and MT) occurs only at high MT frequencies. The 1-D optimization scheme is easily implemented because of the common physical parameter, that of electrical resistivity, and the common forward model, that of MT. The objective function to be minimized is composed of three terms: a term related to the TDEM converted data, a term related to the MT data and a term related to the structure according to the Occam-like scheme (see Godio and Santilano 2018). Furthermore, the static shift *S* is an element of the model vector, i.e., a parameter optimized by PSO. It is included in the objective function and adopted as multiplier of the MT observed data. The best solution provides the smoothest model and the proper static shift value in accordance with the reference TDEM-converted curve.

**Fig. 11:**
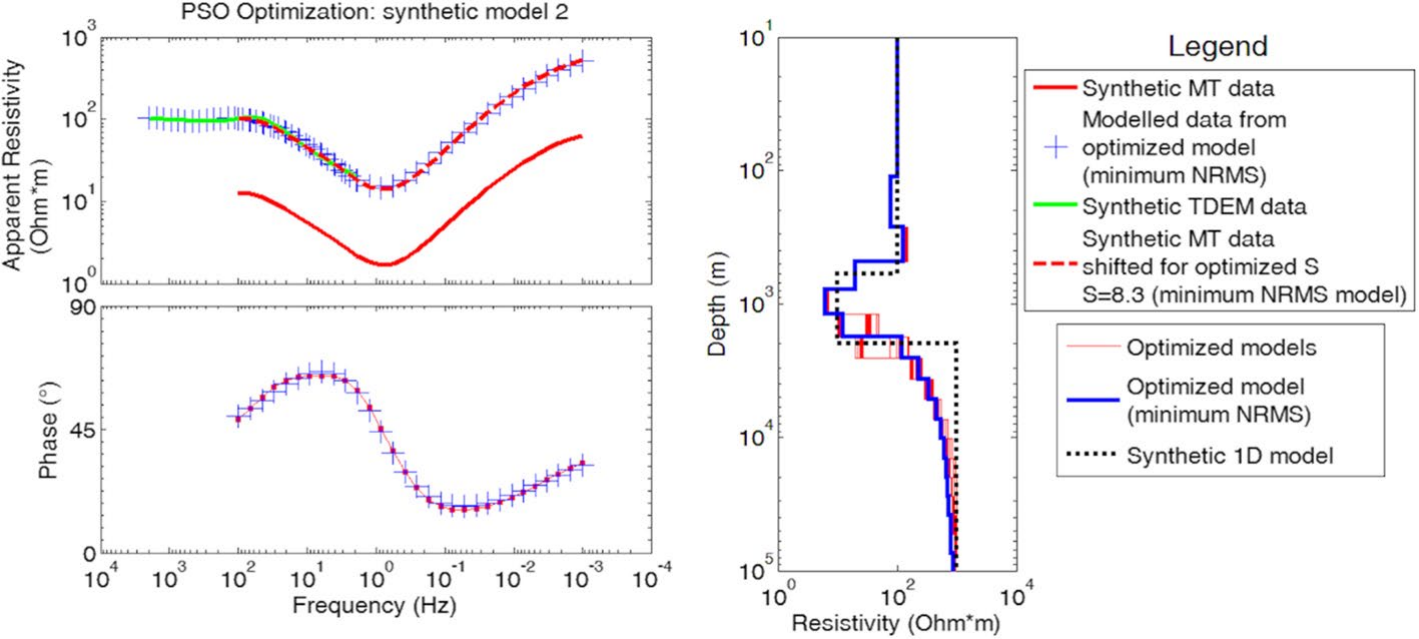
The PSO optimization of a synthetic model of TDEM and MT data (black dotted line in the right-side plot) from Santilano et al. (2018). The resulting 25 models are shown in red, and the model with the minimum NRMSE is shown in blue. On the left, the theoretical MT data are shown for the minimum NRMSE model (blue crosses) and compared with the synthetic MT (red line) and TDEM (green line) data. The MT apparent resistivity curve multiplied by the optimized factor *S* for static shift correction is marked with red dashed line

The approach is tested on a synthetic model (Fig. 11) and on field data acquired in the Larderello geothermal field (Italy). The PSO input arguments are constant accelerations (cognitive attraction *α*_1_ = 0.75 and social attraction *α*_2_ = 1.75), stopping criterion at 200 iterations, swarm size of 300 particles, random initialization and resistivity search space bounded between 1 and 2000 Ωm. The same PSO setup was repeated 25 times (or trials).

The main contribution of this work is that PSO was validated to correct, retrieve and remove the static shift from MT data by simultaneous analysis of MT and TDEM data. Furthermore, running several PSO trials with the same settings allows the robustness of the retrieved static-shift value to be assessed.

## PSO of other Geophysical Data

### Gravity

The gravimetric method is adopted in applied geophysics to study the delineation of sedimentary basins in hydrocarbon and mineral exploration, hydrogeology, fault investigation, glaciology, etc. (Telford et al. 1976). The gravity inverse problem is linear if the geometry is provided and the density estimated or, conversely, nonlinear if the geometry is the unknown and the density is assumed. The nonlinear inverse problem is commonly solved by means of iterative linearization using the Levenberg–Marquardt algorithm (L–M), whose solution is however strongly influenced by the initial model (prior information) and suffers from a correct uncertainty analysis.

PSO has been applied to both synthetic and field gravity data for 2-D (Yuan et al. 2009; Pallero et al. 2015, 2021; Darisma et al. 2017; Essa and Munschy 2019; Anderson et al. 2020; Essa and Géraud 2020; Essa et al. 2021) and 3-D interpretations (Pallero et al. 2017; Jamasb et al. 2019).

The first application of PSO to 2-D synthetic data with and without noise (Yuan et al. 2009) adopts a standard PSO algorithm (constant accelerations) and random initialization. The acceleration value is 2 for both cognitive and social accelerations, while the inertia weight is 1. PSO proved to outperform the L–M method in terms of data fitting and independence from the starting model and to outperform GA in terms of accuracy and converging time. Gravity modeling in 2-D and the specific objective function for gravity data are well explained in Darisma et al. (2017). They perform a sensitivity analysis on the influence of the PSO input parameters on the model solution and choose the constant values of 0.79, 0.79 and 0.86 for the inertia weight, cognitive and social accelerations, respectively. The best solution is achieved with the swarm size equal to 2 times the unknowns, but values up to 5 times are tested.

Pallero et al. (2015) adopt a full family of PSO optimizers for the 2-D gravity inversion and model appraisal (uncertainty assessment) of basement relief in sedimentary basins. The family of PSO algorithms includes Generalized PSO (GPSO), CC-PSO and CP-PSO, which were firstly presented in Fernández Martínez et al. (2010a), and new optimizers called RR-PSO and PP-PSO. While CP- and PP-PSO are explorative algorithms, RR-PSO has a good balance between exploration and exploitation of the search space. For further details on the family of PSO, the reader is referred to the cited works (Fernández Martínez et al. 2010a, 2012; Pallero et al. 2015). The cloud version of the adopted algorithms is based on the “free-parameter tuning philosophy”, which automatically choses the input parameters (inertia, local and global accelerations) from the regions of the search space that are more stable. The full family of PSO optimizers is applied to a gravimetric profile from Atacama Desert in north Chile. Their PSO algorithms perform a fast inversion and uncertainty assessment of the gravimetric model using a sampling instead of an optimizing procedure. They show the 10% equivalence region of the best PSO model and conclude that “*inversion and uncertainty analysis (solution appraisal) must always go hand in hand*”. The proposed method appears as a powerful tool for an accurate estimation of basement relief of sedimentary basins, taking into account the actual topography of the gravity observations.

Another study of 2-D synthetic and field gravity data has been recently published by Anderson et al. (2020) to model 2-D vertical faults and to calculate fault parameters (depth, amplitude factor and origin of the fault trace). They adopt standard PSO with constant accelerations (equal to 2) and random initialization. Even though using classical PSO, the proposed method shows fast convergence and does not require a priori initialization.

**Fig. 12:**
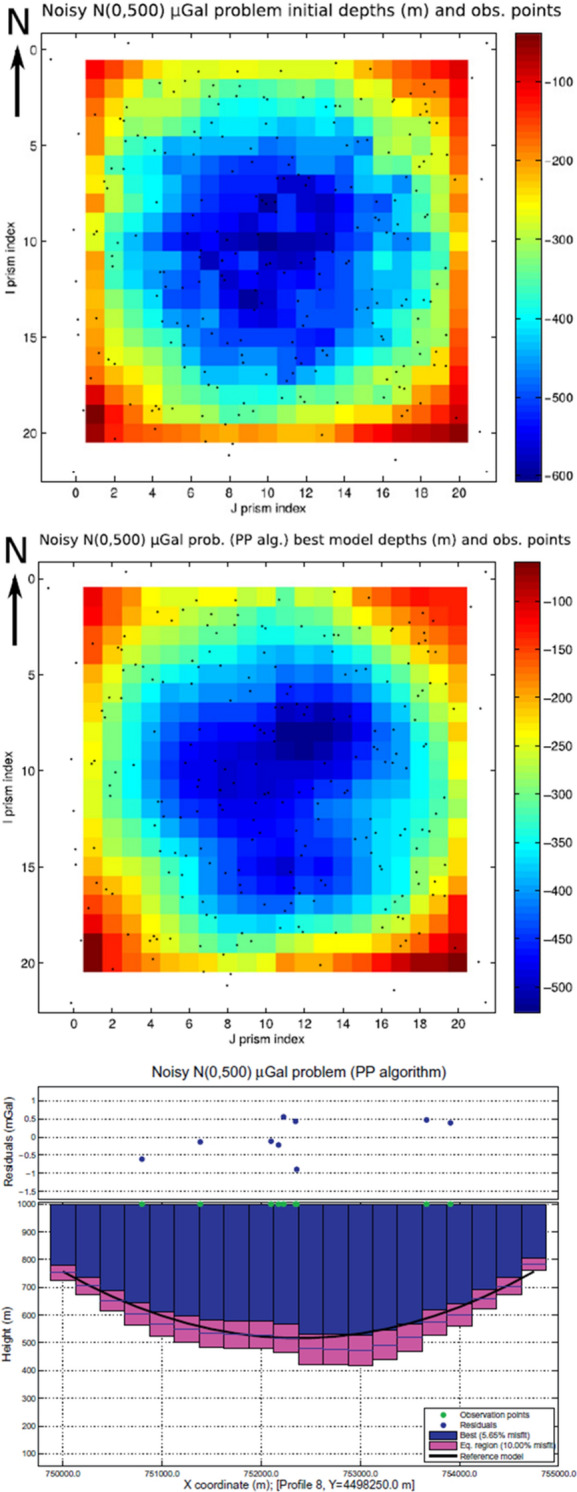
Synthetic case study with noisy gravimetric data from Pallero et al. (2017). Top: true model. Middle: best model after PP-PSO. Bottom: WE profile from the best solution showing the true depth (black curve), the best solution depth (blue prisms), the 10% misfit solutions (pink prisms), the observation points (green dots) and the residuals (black dots)

The common nonlinear approach for the 3-D gravity problem consists in modeling the basin as a set of regular prisms whose unknown is either the density contrast or depths. Pallero et al. (2017) propose the application of a family of PSO optimizers to 3-D gravity inversion in sedimentary basins in order to estimate the height of the prisms given the density contrast (fixed or variable) between the sediments and the basement. The coefficients of the regional trend affecting the observed data are treated as additional unknowns. A family of global PSO optimizers is applied to both synthetic and field case studies: GPSO, CC-PSO, CP-PSO, PP-PSO and RR-PSO (Pallero et al. 2015). These algorithms deploy free-parameter tuning, meaning that inertia, cognitive and social accelerations change for every particle of the swarm. These three coefficients are indeed constant with the iterations, but that are chosen close to the limit of second order stability of each PSO member to ensure a balance between exploration and exploitation of the search space. It is demonstrated that CP-PSO has a high exploratory character, while RR-PSO and PP-PSO have a more exploitative character, yielding a constant decreasing of the relative error. CC-PSO and GPSO instead reach stabilization in the region of low relative error (2%). The PP-PSO proves to be the best PSO variant applied to synthetic data with Gaussian noise since the best solution and the 10% equivalent region match the true model (see Fig. 12). This demonstrates that different PSO algorithms ensure different levels of solution convergence. Pallero et al. (2017) provide a valid method to solve 3-D gravity inversion and quantify the uncertainty (model appraisal) of the inverted depth model.

Jamasb et al. (2019) present a hybrid PSO that adopts evolution strategies in order to speed up the converging time to solve the “high-dimensional” 3-D nonlinear gravity inversion problem (note that the model parameters are around 10). The proposed hybrid PSO outperforms classical PSO in real field applications for the estimation of the thickness of a sedimentary cover without a priori assumptions.

Recently, Pallero et al. (2021) presented the MATLAB software package “GRAVPSO2D” for the 2-D inversion of gravity data. They focus on the nonlinear problem of the estimation of the basement geometry in sedimentary basins and use a broad family of PSO optimizers. The source codes and the package are freely available and can represent an important tool to boost the adoption of PSO by the geophysical research community. Besides the synthetic and real case studies that prove the efficiency of the code, Pallero et al. (2021) present a full description of the implemented methodologies, the possible workflows, as well as the guidelines for running the optimization. Conceptually, GRAVPSO2D implies the juxtaposition of rectangles along the profile, whose top represents the topography, whereas the bottom corresponds to the sediments-basement interface. The input data for the inversion are the Bouguer gravity anomalies observed along the profile. The anomaly generated over each observation point by a 2-D model composed of juxtaposed rectangles is computed with a forward operator as a function of the rectangles and their position with respect to the point and as a function of the rectangles bottom depth. The algorithm considers the latter, i.e., the elevation of the rectangles bottom, as the problem unknowns. The package allows the use of constant or variable density contrasts (vertically or horizontally). The contribution of regional trends can also be modelled. The tool also performs the uncertainty analysis on the resulting model(s) by showing the inversion residuals, the best model and the equivalence region of the solutions. Moreover, the authors propose the analysis of the cumulative distribution of those models sampled during all the iterations and falling within a certain percentage of misfit tolerance.

### Magnetic

The solution of the magnetic inverse problem is addressed according to several approaches. The basic analysis focuses on the retrieval of the main geometrical factors of the target, such as the shape, depth and magnetization properties (Abdelrahman et al. 2003, 2007; Abdelrahman and Essa 2005, 2015). Conventional methods to retrieve the model parameters of special features, such as thin sheets or dykes, are based on the least-square minimization approach (e.g., Abdelrahman and Sharafeldin 1996; Abdelrahman et al. 2009).

Stochastic approaches have recently been introduced for the nonlinear inversion of magnetic anomalies caused by different geological features such as faults, thin dikes and spheres (Asfahani and Tlas 2007). Metaheuristic methods have been adopted to interpret the magnetic anomalies caused by simple features. A global optimization algorithm (the very fast SA) was developed to interpret gravity and magnetic anomalies over thin sheet-type structures for mineral exploration (Biswas 2016) and to interpret the total gradient of gravity and magnetic anomalies caused by thin dyke-like structure embedded in the shallow and deeper subsurface (Biswas et al. 2017). The model parameters are the amplitude coefficient, the exact origin of causative source, depth and shape factors. The GA is adopted to solve the general equation of magnetic anomalies in Kaftan (2017).

**Fig. 13:**
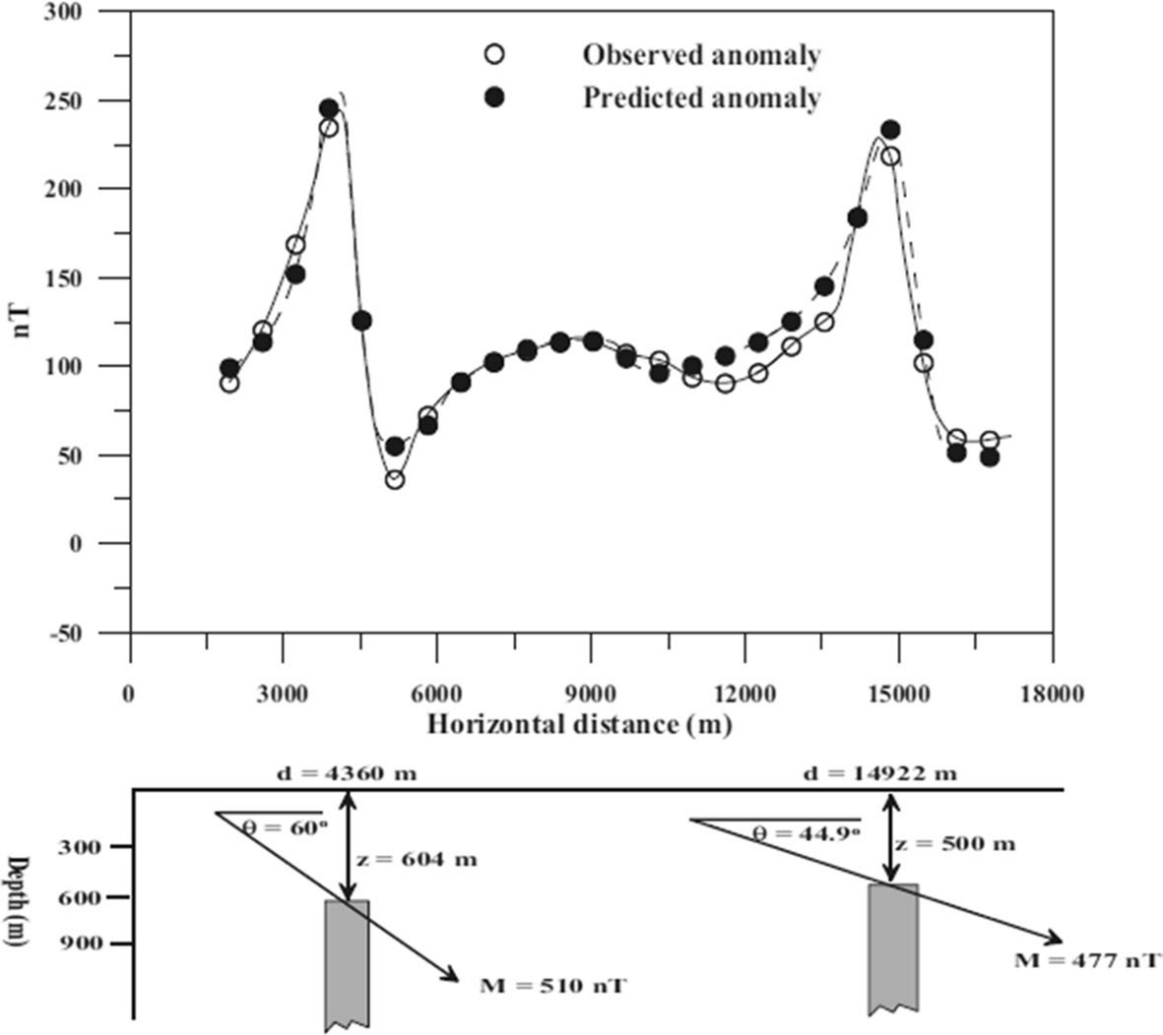
Field case study from Hamrawein area (Egypt) in Essa and Elhussein (2020). Top: the data fitting between observed data (white circles) and PSO-predicted response (black circles). Bottom: cross-section of the model

The PSO algorithm has recently been proposed for the interpretation of magnetic anomalies with simple geometry (e.g., isolated sources embedded in the subsurface). Essa and Elhussein (2018) test both noise-free and noisy synthetic and real-field data with a standard PSO algorithm. PSO demonstrates speed of convergence, solution stability, applicability for a fast evaluation of the best model-parameter values and a chance to provide the initial model for conventional least-square inversion. Essa and Elhussein (2020) apply classical PSO to several synthetic and real case studies for mineral exploration. To remove regional anomalies up to the third-order, they optimize the second moving average residual magnetic anomalies for different window lengths (*s*-values). The model parameters are the depth of the body, the amplitude coefficient, the angle of magnetization, the shape factor and the horizontal coordinates of the source along the profile. PSO input arguments are constant: 0.8 for inertia weight and 2 for the accelerations. For the field data set from Hamrawein area (Egypt), the data fitting between observed data and PSO-predicted response in shown in Fig. 13 with a final RMS value of 11.4 nT after 400 PSO iterations. The PSO outcome is also in good agreement with known geological and geophysical information. Even though Essa and Elhussein (2020) adopt a standard PSO algorithm (constant inertia and accelerations), they outline the PSO efficient computation and the solution robustness with respect to GA and SA.

Complex magnetic anomalies due to multiple sources are investigated in Liu et al. (2018). They adopt standard PSO with smoothing for the particle velocity to solve the 2-D inversion of magnetic data and recover the magnetization intensity of the subsurface. The PSO input parameters are tested with a comprehensive sensitivity analysis on the influence of inertia and accelerations on the convergence curves of PSO (the RMS trend as a function of the iterations). The most appropriate input arguments they find for a stable and fast-convergence optimization of magnetic data are linear decreasing inertia weight (from 0.96 to 0.6) and constant accelerations (equal to 2). This PSO setup is applied to both synthetic and field examples from iron ore deposits. The 2-D model domain is divided into 800 regular cells and the number of particles is 20. The iterations converged in 200 to 400 iterations, depending on the case study they investigate. The uncertainty analysis of the PSO models and their comparison with drill-hole information demonstrate the validity of the proposed methodology.

The nonlinear inversion of magnetic anomalies from 3-D prismatic bodies has been poorly addressed using metaheuristics. An example is the application of the differential evolution algorithm, a population-based evolutionary algorithm (Balkaya et al. 2017).

### Self-potential

The self-potential (SP) method is widely referred to as the “ugly duckling” in geophysics as it involves simple procedures in both data acquisition and processing (Nyquist and Corry 2002). The data acquisition involves simple equipment consisting in non-polarizing electrodes, voltmeter or data logger and electrical cables. No artificial source is required. This makes the method cheap and easy to be applied in multiple fields, not only for geo-exploration but also for monitoring dynamic processes. As the self-potentials are related to groundwater flow, temperature gradients, chemical processes and biological effects, the method is widely adopted in environmental and engineering investigations (Vichabian and Morgan 2002), hydrology and hydrogeology (Jouniaux et al. 2009), geothermal exploration (Corwin and Hoover 1979) and other fields. Thanks to its simplicity, it is also suitable to describe the meaning of forward modeling and inversion procedures, that could be then extrapolated to other geophysical methods.

Focusing on the interpretation of SP signals, the most recent updates cover mining exploration and monitoring. A global optimization method based on a Genetic-Price hybrid Algorithm (GPA) has been proposed for identifying the source parameters of SP anomalies (Di Maio et al. 2019). The approach leads to the interpretation of simple polarized structures, such as spheres, vertical or horizontal cylinders and inclined sheets. Singh et al. (2019) discuss the use of GA on SP data for the prediction of coal seam fire in India. The SP anomaly is mainly caused by the thermoelectric effect of the temperature gradient observed in the coal fires. Their results estimate the depth of the fire regions and other geometrical factors to characterize the fire regions. Göktürkler and Balkaya (2012) compare the performance of GA, SA and PSO to invert SP anomalies originated by polarized bodies with simple geometries. Field tests are a copper belt (India), graphite deposits (Germany) and metallic sulfide (Turkey).

**Fig. 14:**
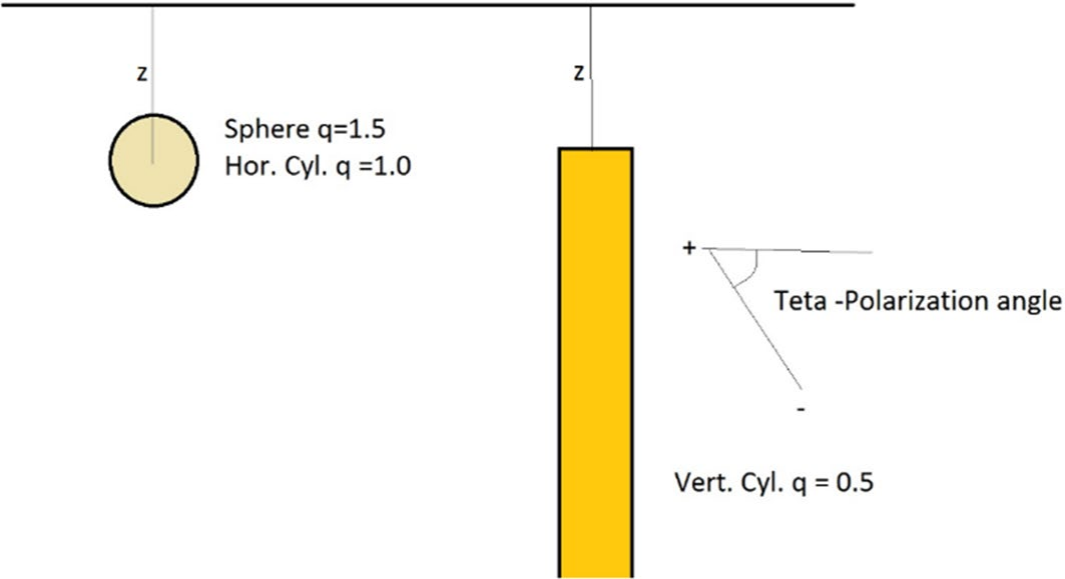
Vertical section of the subsoil with the shape factor *q* for a sphere and a cylinder

The optimization procedure considers the general equation of the SP anomaly, which relates the voltage to the geometrical factors of simple targets. Following Yüngül (1950) and Bhattacharya and Roy (1981), the SP anomaly expression, *V*, produced by most polarized structures is given by the following:

13 $$V\left( {x_{i} , z, \theta , q} \right) = K \frac{{x_{i} \cos \theta + z \sin \theta }}{{\left( {x_{i}^{2} + z^{2} } \right)^{q} }},\quad i = 1, 2, 3, \ldots , N$$

where *z* is the depth of the body, *x*_*i*_ the location coordinate, *K* the electric dipole moment, *θ* the polarization angle and *q* the shape factor. The shape factors for a sphere, horizontal cylinder, and a semi-infinite vertical cylinder are 1.5, 1.0, and 0.5, respectively (see Fig. 14).

PSO can be used for quantitative interpretation of SP data (Santos 2010; Essa 2019, 2020). For a single target, the optimization procedure is reduced to a trivial estimate of the model parameters, such as the depth of the source, the distance from the origin, the electric dipole moment, polarization angle, shape factor and regional coefficients. Pekşen et al. (2011) investigates the reliability and the performance of PSO in solving the SP problem and introduces a customized statistical analysis to reduce the ambiguities of the optimization procedure.

**Fig. 15:**
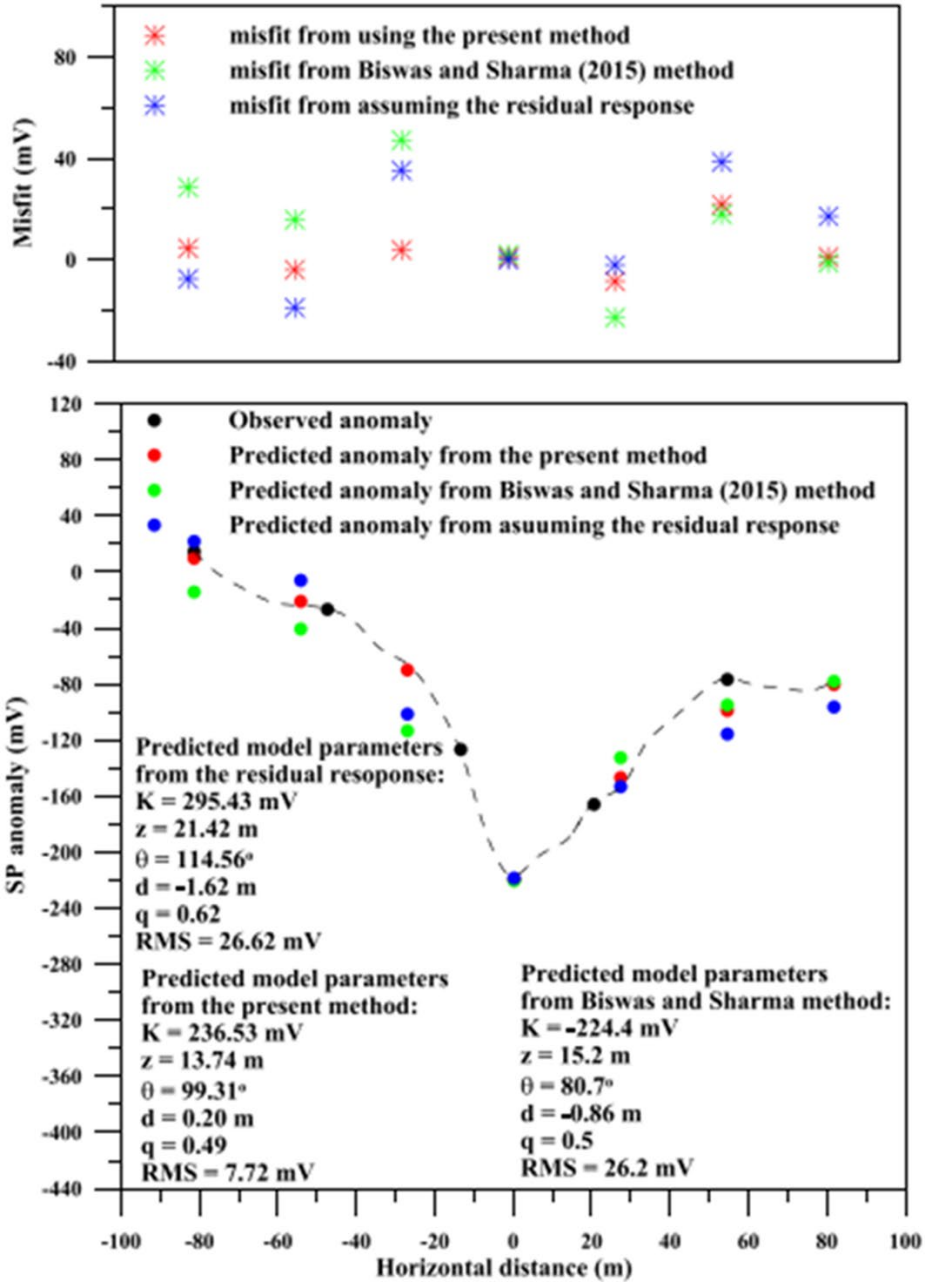
Result of PSO applied to an SP anomaly over the Malachite Mine (USA) as interpreted by Essa (2019). Top: misfit between the observed and predicted anomalies (red stars are from Essa 2019). Bottom: observed (black) against predicted SP response using PSO (red), very fast SA (green) and assuming residual response (blue)

A well-known literature case study is the interpretation of the SP anomaly in the Malachite Mine, Colorado, USA (Dobrin and Savit 1960, p. 426). PSO has recently been applied to the second moving-average residual SP anomalies of the Malachite Mine to determine the five above-mentioned parameters for various *s*-values (Essa 2019). This SP profile is shown in Fig. 15 together with the misfit between the observed and predicted anomalies. The values for inertia weight and accelerations are constant and equal to 0.8 and 2, respectively. The final parameter values for the best PSO solution are: *K* = − 236.53 mV; *θ* = 99.31°; *z* = 13.74 m; *x* = 0.2 m; *q* = 0.49. The final RMSE is 7.72 mV.

### Direct Current

One of the first applications of PSO to electrical methods has been published by Fernández Martínez et al. (2010a). They apply PSO to the VES inverse problem and test its performance with a 6-layer parametrization for a real case study related to seawater intrusion in a coastal aquifer in Spain. They examine a family of PSO algorithms with these setups for inertia weight, cognitive and social accelerations, respectively: (0.687, 2.25, 1.125) for GPSO, (0.629, 2.67, 1.33) for CC-PSO, (0.545, 3.27, 1.64) for CP-PSO. These values are proved to ensure closeness to the second order stability region and to avoid entrapment in local minima regions. They observe that for the VES problem the CP-PSO variant has a very god convergence rate and a good balance between explorative and exploitative behavior. The family of PSO variants is compared to other global optimization algorithms (binary GA and SA) to check their respective convergences given the same search space and number of particles (200) and iterations (100). It is demonstrated that the PSO family variants converge in the low-misfit zone (5–6%) after around 10–20 iterations, while GA and SA do not reach it in 100 iterations.

Pekşen et al. (2014) propose a PSO method to solve the problem of a layered anisotropic earth model and estimate vertical and horizontal resistivity (2-D) and layer thickness. They adopt standard PSO with input parameters as suggested in previous applications (Fernández Martínez et al. 2010a). They demonstrate the suitability of the method on noise-free and noisy synthetic and field DC data. The most interesting part of the work is the a posteriori analysis of the PSO outcome in terms of: general behavior of the model parameters during the iterations, frequency distribution and probability density function of the parameters. To reduce the ambiguity of the result, only the anisotropic model parameters whose probability density function is higher than 95% are selected. Other PSO applications to VES data regard the joint interpretation of VES and TDEM data (Cheng et al. 2015; Pace et al. 2019b).

### Seismic and Ground Penetrating Radar

We distinguish the application of PSO to data processing and interpretation of seismic data, according to the distinction between induced seismicity and active seismic for exploration and geotechnical characterization.

- *Seismic* Rayleigh waves have been increasingly used as an appealing tool to obtain near-surface shear-wave velocity profiles. Inversion of Rayleigh wave dispersion curves is challenging for most local-search methods due to its high nonlinearity and multimodality. A Rayleigh-wave dispersion-curve inversion scheme based on PSO has been developed by Song et al. (2012) for the inversion of noise-free and contaminated field data sets. They adopt the PSO variant with the constriction factor in order to enhance local search and convergence (Shi and Eberhart 1998). An accurate inspection of the PSO results is provided by means of histograms of the inverted model-parameters belonging to the low-misfit region with less than 15% of relative error. The histograms have symmetric shape and contain the true value (of the synthetic model) within the high density area, thus proving that PSO performs a good posterior sampling of the equivalence region. Moreover, the efficiency and stability of PSO is examined by means of a comparative analysis with GA and MC. Results from synthetic and field data demonstrate that PSO provides better performances than GA and MC in terms of quality of the solution, speed of convergence and computational efforts. The main advantages of PSO consist in the location of the low misfit region and the easiness of implementation.
  Swarm intelligence has proved to be effective as inversion method for seismic wavelet analysis (Yuan et al. 2009). The feasibility and accuracy of PSO has been tested on a theoretical model of wavelet, simulating the response of a layered medium. One of the most interesting findings is the great stability of the PSO with respect to noise. While PSO outperforms GA with the best performance in terms of accuracy and convergence speed, PSO and SA have a comparative accuracy and convergence speed even though the final misfit between the original and estimated wavelets is higher in SA than in PSO.
- *Microseismic* Lagos and Velis (2018) detect microseismic events associated to hydraulic fracturing by using very fast SA (VFSA) and PSO and compare them to the classical grid search (GS). Their PSO workflow merges into an automated process the different steps that lead to the microseismic events location starting from raw data. The first step of the workflow deals with the automatic detection, denoising and identification of the P- and S-waves. The second step estimates the corresponding back-azimuths using polarization information and selects the most reliable estimate to restrict the search space of model parameters (microseismic source coordinates). Finally, the location of the events is performed by solving a nonlinear optimization problem using the VFSA and PSO algorithms for 2-D and 3-D usual scenarios of hydraulic fracturing. The main conclusion highlights the advantage of using either VFSA or PSO instead of GS in terms of computation speed-up.

## Performing Joint Inversion with PSO

Multi-objective PSO can solve the nonlinear joint inversion of different geophysical data sets. The MOPSO algorithm has revealed a number of attractive features: a single tool to tackle multiple data sets, a set of final models without multiple conflicting solutions and an effective insight in the trade-off meaning of the final solutions. The general theory of optimization of multi-objective problems is based on the concept of Pareto optimality. The best trade-off solutions and their range are usually identified as final solutions because of the Pareto dominance. Moreover, the shape of the Pareto Front (*PF*) provides insight into the compatibility between different geophysical data sets.

Initial attempts to solve joint optimization by means of PSO have actually simplified the multi-objective problem to a single-objective one. As an example, Cheng et al. (2015) apply PSO to a whole forward process synchronized between TEM and DC methods. Basically, the authors build a single data vector including the two different (modelled or observed) data sets, as well as a single residual vector that computes the objective function. The same weight is used for the two datasets. This simplistic but effective procedure is applied to a civil engineering case study in a coalmine in China.

The adoption of a MOPSO scheme avoids both the simplification of a multi-objective problem and the use of weighting factors between different data sets. Another advantage of the multi-objective optimizer is to overcome the intrinsic limitations of each geophysical method. Some representative examples exploit MOPSO to jointly interpret TDEM and VES data (Pace et al. 2019b) and GPR and P-wave seismic travel-times (Paasche and Tronicke 2014). The economic concept of Pareto optimality is adopted to identify the final set of results among the feasible solutions.

**Fig. 16:**
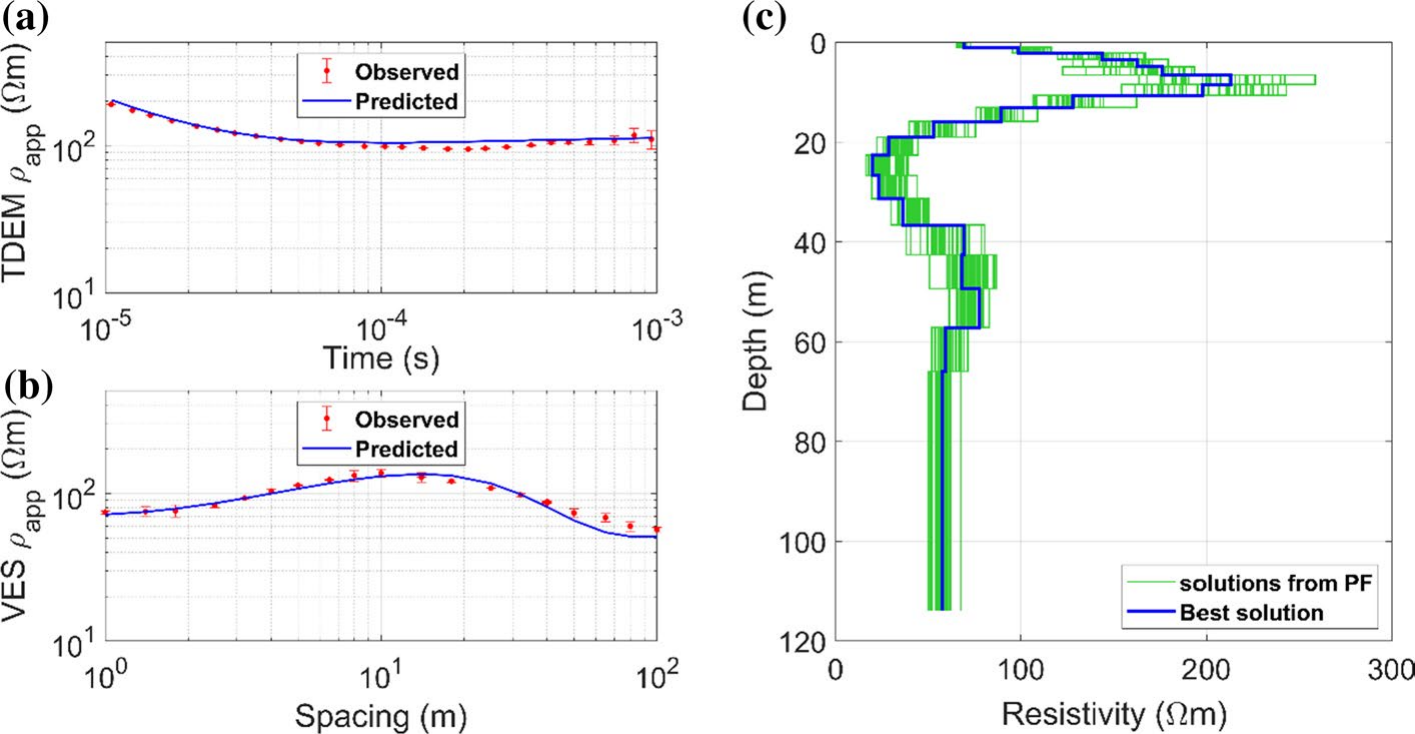
MOPSO applied to field electric and TDEM data from Pace et al. (2019b): observed data (red dots with error bars) and predicted apparent resistivity (blue-line*-ρ*_app_) for TDEM (**a**) and VES (**b**) data; **c** the final resistivity models belonging to the *PF* (green lines) and the best solution marked in blue

In the case study by Pace et al. (2019b), MOPSO is applied to a field data set composed of TDEM and VES soundings over a known stratigraphic setting in Piedmont, Northwest Italy. The physical parameter to be optimized in a layered-model is the same for the two methods, i.e., the electrical resistivity. The TDEM data were acquired in the range 10^–5^ s and 10^–3^ s using a coincident-loop configuration with a 50-m-long loop. The VES data were collected according to a Schlumberger array and deploying a 100-m maximum half-spacing of the current electrodes. The model is discretized into 19 layers and an Occam-like minimization is implemented to obtain smooth models (objective function like Eq. 11). The optimal Lagrange multiplier of the objective function is the same for both TDEM and VES components following the L-curve criterion. The boundaries of the solution search space are the minimum and maximum resistivity values of 1 Ωm and 500 Ωm, respectively. The MOPSO algorithm with time-varying accelerations coefficients is adopted: cognitive accelerations linearly decreasing from 2 to 0.5, social acceleration linearly increasing from 0.5 to 2, inertia weight linearly decreasing from 0.9 to 0.4. The MOPSO algorithm ran for 1000 iterations, giving in the end the family of Pareto-optimal solutions plotted in Fig. 16c. The data fitting is shown in Fig. 16a, b for TDEM and VES, respectively. The number of particles forming the swarm is 170, following the rule of thumb to set the swarm size as nine times the unknowns*.* The solutions drawn from the *PF* are depicted in green, while the blue line corresponds to the solution with the minimum value for both the components of the objective function. The resistivity model reveals a resistive layer of about 200 Ωm in the shallow subsurface, till 10 m of depth. A conductive region of less than 50 Ωm appears from a depth of about 20–40 m, while, at higher depths, the resistivity increases to 77 Ωm. The PSO resistivity model is supported by the geological information derived from a borehole located very close to the investigated site. A major result is the comparison between the resistivity model from MOPSO and the models from separate optimizations using single-objective PSO (not shown here). Indeed, the model resulting from PSO applied to only TDEM underestimates the shallow resistive layer due to the intrinsic limitation of EM methods that are more sensitive to conductors rather than to resistors. Conversely, the model resulting from PSO applied to only VES images the shallow resistive layer and underestimates the deep conductors. The resistivity model obtained from MOPSO correctly images the two conductive and resistive structures and compares well with the stratigraphic well log (not shown here).

**Fig. 17:**
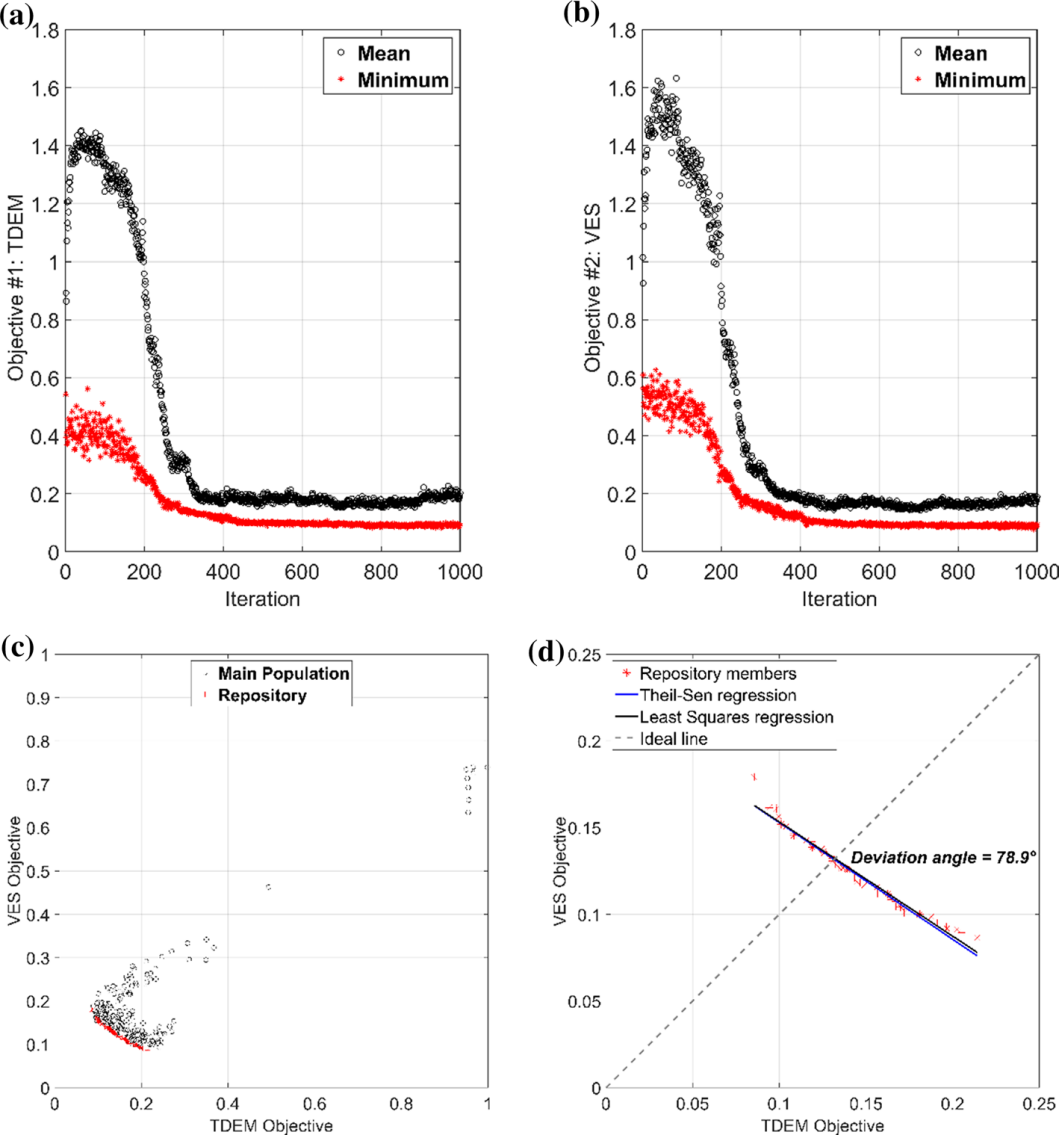
The MOPSO performance as in Pace et al. (2019b). The evolution of the TDEM (**a**) and VES (**b**) components of the objective function from the first to the last iteration for the best particle (red stars) and the remaining ones (black circles); **c** the 2-D space of the objective function (TDEM and VES components) at the last iteration: the red symbols identify the *PF* and the black circles the objective-function values assumed by the other solutions; **d** the intersection between the ideal line (grey dashed) and the Theil-Sen regression line (blue) or the least-square regression line (black) identifies the deviation angle *α*

Figure 17a, b shows the simultaneous minimization of the two components (TDEM and VES, respectively) of the objective function from the first to the last iteration. The red stars refer to the particles with the minimum objective-function component, while the black circles to the mean value. Figure 17c depicts the 2-D objective space at the final iteration. The *PF* is marked in red, while the black circles represent the objective function of the remaining solutions. The *PF* can be evaluated by three metrics. The repository index (*RI*), that measures the ratio of the non-dominated solutions with respect the population size, was 21.5%. The spacing (*SP*), that measures the solution distribution throughout the *PF*, was 0.0041. The deviation angle *α* between the ideal line and the Theil-Sen-regression line over the non-dominated solutions was 78.9°. Figure 17d zooms in the PF and shows the deviation angle between the grey-dashed ideal line and the Theil-Sen-regression blue line. Since *α* is higher than 45° and the *PF* is not symmetric, a slight conflict between TDEM and VES can be inferred (Dal Moro and Pipan 2007; Schnaidt et al. 2018).

**Fig. 18:**
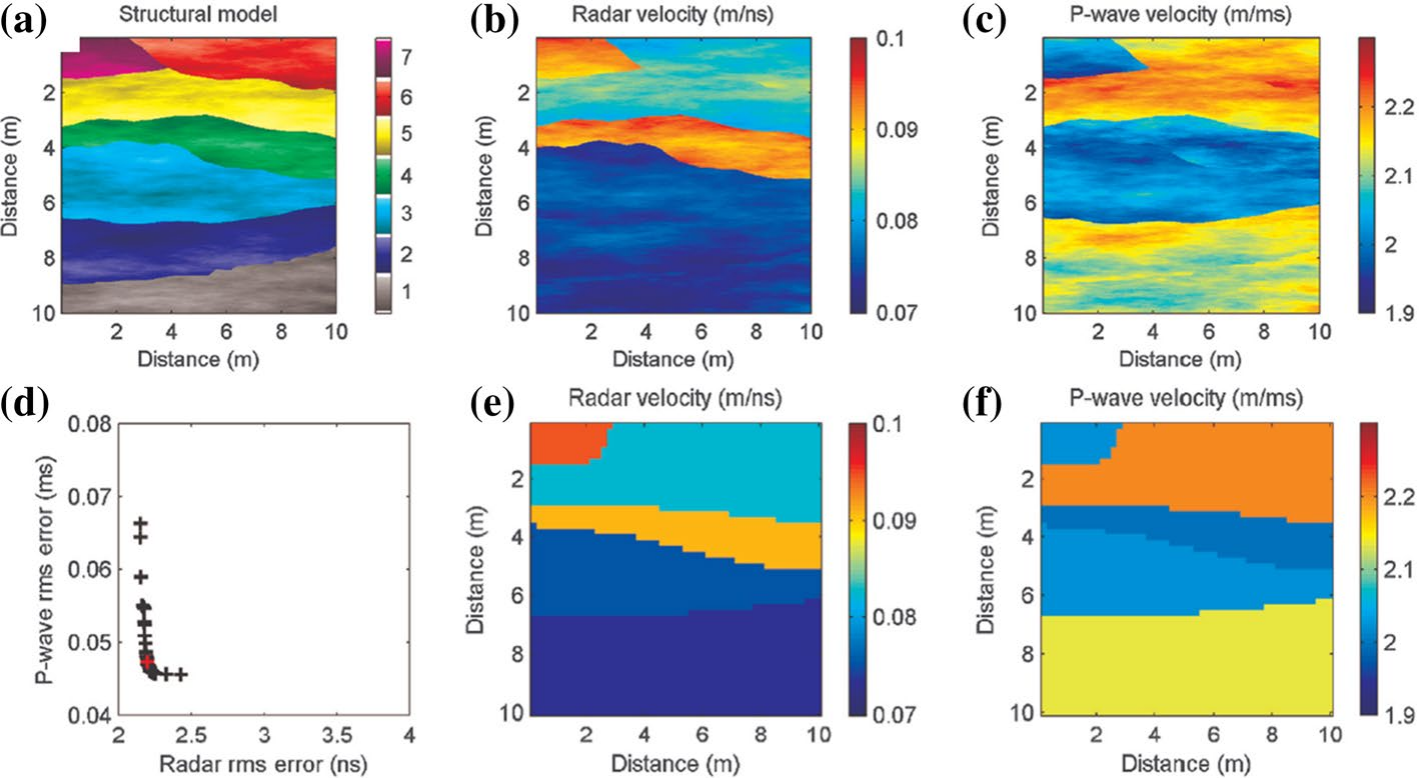
Pareto-dominance-based nonlinear joint inversion using MOPSO of radar and P-wave velocity from Paasche and Tronicke (2014); **a** structural model composed of seven layers; synthetic data are generated from georadar (**b**) and P-wave (**c**) velocity fields; **d** the Pareto Front from MOPSO is composed of 39 optimal solutions (crosses) after 1000 iterations; the red symbol denotes the position in the Pareto Front of the optimal solutions in **e** and **f**

In addition to the Pareto dominance adopted in MOPSO, Paasche and Tronicke (2014) test a hybrid approach to jointly invert synthetic cross-hole tomographic datasets composed of radar and P-wave travel-times. This approach is based on a first Pareto-dominance-based nonlinear joint inversion and then a linear-aggregation-based nonlinear joint inversion. The starting model consists of seven layers exhibiting spatial heterogeneity (Fig. 18a). The layers are associated to petro-physical parameters to model the propagation of geo-radar and seismic wave-field velocity (Fig. 18b, c, respectively). The edges of the model represent boreholes, with a 0.25 m spacing of equally distributed sources and receivers. The proposed layer-based parameterization of the model domain results from a deterministic zonal cooperative inversion (ZCI) to support the finding of an adequate minimal complexity (Paasche and Tronicke 2007). The number of model parameters to be optimized is 26. The database is first jointly inverted using the MOPSO algorithm exploiting the Pareto dominance. In order to rank the available Pareto optimal models, the authors exploit the concept proposed by Balling (2003) based on game theory. The approach considers the objective functions as competing agents with conflicting interests in a zero-sum game (Raghavan 1994). The swarm is initialized with 48 particles, apparently without following the rule of thumb to size the swarm at least nine times the unknowns. At the end of the multi-objective optimization the final Pareto set of optimal solutions had 39 solutions, as shown in the Pareto front of Fig. 18d with crosses. The red symbol depicts in the search space the Pareto optimal solution drawn in Fig. 18e, f for radar and P-wave velocity, respectively.

The test accomplished in Paasche and Tronicke (2014) provides for a further transformation of the MO optimization problem into a single-objective one based on the idea of aggregation-based solutions (e.g., Parsopoulos and Vrahatis 2002). This additional scheme is appended at the end of the MOPSO algorithm and exploits the set of solutions already computed. The aim is to find the solutions in the vicinity of the point of maximal curvature on the Pareto Front found by MOPSO. The radar and seismic objective functions are scaled according to the RMS errors at the point of maximal curvature found by the Pareto-dominance-based approach. The simplification of the MO optimization problem into a single-objective problem by linear aggregation implies, in terms of computational effort, an inefficient identification of several mutually independent solutions of the problem. The optimization is repeated several times. The result comprises 150 radar and seismic velocity models. Five models out of 150 are non-dominated and represent a very short segment of the Pareto Front. Paasche and Tronicke (2014) conclude that the Pareto-dominance-based approach is able to efficiently detect the non-dominated solutions with some risk of mutual model dependency in the final set of solutions, whereas the linear-aggregation approach turns out to be robust but time consuming and hence inefficient.

## Best Practices with PSO

We here propose practical and useful guidelines to perform stochastic inverse modeling of any kind of geophysical data set for the benefit of PSO users. The fundamental rules of thumb to run PSO can be schematically summarized as follows:

1. Evaluate the mathematical complexity and computational load of the geophysical forward problem. The complexity influences the choice of the PSO variant in terms of balance between explorative and exploitative behavior. The expected runtime influences the optimization settings such as the number of iterations, trials and particles.
2. Choice of the PSO variant: an updated overview of the most recent PSO variants (as those listed in Sect. 2.2) can be of help to choose the algorithm that best fits the specific geophysical problem in terms of search space exploration and solution convergence. For simple problems the classical PSO can be appropriate enough, but for increasing complexity (in the number of unknowns or equivalent solutions) HPSO-TVAC or similar variants are highly recommended.
3. Model discretization: the 1-D, 2-D or 3-D geophysical model should be appropriately discretized in order to, on one hand, obey the under-determined condition of the inverse problem, and, on the other hand, to avoid excessive computational load due to numerous unknowns. It is also possible to use basis functions in order to decrease the number of parameters and to unburden the computational load of the forward modeling. For example, in Aleardi (2019), orthogonal Legendre polynomials are used as basis functions to reparameterize the model space. The expansion coefficients associated with each Legendre polynomial are determined via PSO.
4. Input arguments (see Sect. 2.2): they are problem-dependent and imply a good knowledge of the mathematics and physics of the problem.
  The accelerations coefficients *α*_1_ and *α*_2_ have to be chosen following either a sensitivity analysis or literature findings in order to balance exploration and exploitation.
  The stopping criterion/-a may vary according to the objective-function trend as a function of the iterations.
  The number of particles *N* depends on the model discretization as it is proportional to the number of unknowns. As a rule of thumb, the number of particles of the swarm could be selected as 9 times the unknowns;
  The boundaries of the search space have to be tailored, namely, far larger than the expected values of the solution to ensure an effective exploration behavior. Too wide search space can result in unnecessary computation. If the final solution is stuck to one of the boundaries, it means that it should be enlarged.
5. Formulation of the objective function with, if preferred, the model regularization (e.g., “Occam’s-like optimization”) and choice of the Lagrange multiplier;
6. External constraints (non-obligatory): the random initialization of the model solutions is the key factor of PSO. However, in case of good reliability of external information potentially constraining the solution, a portion of the swarm can be influenced in the global search. This is done by giving an initial value (a priori information) to the position of a subset of particles at the first iteration. External constraints can also be adopted to perform sensitivity tests as a posteriori assessment of the uncertainty of the final solution.
7. Parallelization of the code: it is recommended when dealing with complex forward routines but it is subject to the availability of adequate computational resources. Multi-core workstations or HPC clusters remarkably speed up the computation.
8. When PSO is running, check for effective minimization of the objective function with the iterations.
9. A posteriori evaluation of the PSO outcome in terms of:
  coverage of the search space: if the distribution of the initial positions of the particles is not dense, increase the number of particles. Otherwise, *N* can be decreased to unburden the computation. If at the early stages the whole swarm occupies a small region, maybe the search-space boundaries should be changed (enlarged or narrowed).
  minimization trend of the objective function: if the curve is not flat at the end, the maximum number of iterations should be increased and vice versa. The stopping criteria may be adjusted as well.
  solution convergence toward the global best: the particles should swarm (i.e., converge) toward the global best at the final iteration. If it is not verified, exploration is maybe still occurring. In this case, either the number of iterations or the social acceleration (or both) should be increased in order to enhance the convergence toward the best particle.
  uncertainty analysis: the a posteriori probability density function of the sampled model parameters should exhibit a unimodal distribution. The larger is the scattering of the results, the poorer is the quality of the solution. Otherwise, be aware of the solution uncertainty. To implement such analysis is not trivial. Two different approaches have been proposed in the literature. The first approach refers to the work by Pallero et al. (2018) that implies the cumulative analysis of all the models of the swarm for every iteration within a certain percentage of error tolerance. The second approach refers to the works by Godio and Santilano (2018), Santilano et al. (2018) and Amato et al. (2021) and implies the cumulative analysis of the best models (minimum RMSE within an error tolerance) emerged from several PSO trials, that have been run several times with identical settings. Even though the second approach is more computationally demanding than the first, it has the advantage of considering the solutions emerged from several PSO trials that have been run with independent random initializations. The first approach instead draws the final cumulative distribution from all the sampled models (all the iterations of a single trial) falling within a certain misfit tolerance. The difference between these two approaches is substantial, but it does not hinder an effective assessment of the solution.

## Conclusions and Suggestions

The state of the art about geophysical modeling using PSO was presented. We described the main features of the algorithm and the recent advances that have improved the solution of nonlinear problems. This review summarized the most representative applications of the PSO algorithm in geophysics. Some original works were selected from the literature to illustrate the adoption of PSO for the solution of the inverse problem of several geophysical data: EM, gravimetric, magnetic, SP, DC and seismic. We focused on the most recent contributions regarding 1-D, 2-D and 3-D PSO of geophysical data to offer an updated and broad overview of original workflows and valid outcomes the PSO has provided in the scientific literature. Joint optimization of multiple geophysical data sets by means of MOPSO was also presented to highlight the advantage of using a single solver that deploys Pareto optimality to handle different data sets without conflicting solutions.

The selection of the works analyzed in this review encompassed the PSO application to complex inverse problems (such as 2-D MT) requiring intense computational effort and the PSO adoption to solve existing scientific issues, such as the correction of the static shift in MT (Sect. 3). So far, the sole PSO application to 3-D interpretation has been for the gravimetric inverse problem, whose solution was found by testing several variants of the PSO algorithm (Sect. 4.1). A recent contribution to PSO of 2-D gravity data has been the release of a code available for the scientific community. The most attractive features of the PSO examples selected for this review are the accurate tuning of the acceleration coefficients of the PSO equation, the extensive analysis of PSO variants to ensure the stability region, the introduction of code parallelization to overcome the high computational cost and the original approaches to handle multiple data sets. In the interpretation of geophysical data, PSO demonstrates to be helpful to account for the error propagation in the model parameters and for the solution uncertainty.

The present work is consistent with the increasing demand for computational-intelligence methods that solve nonlinear or under-determined problems affected by non-uniqueness of the solution. These problems are frequent in a wide range of scientific fields and may receive further comprehension if a metaheuristic approach is adopted to find the model solution. The works selected in this review are of relevant interest not only for the geoscientists directly involved in processing and interpretation of geophysical data but also for the scientific community involved in any kind of optimization process for solving linear and nonlinear problems related to artificial neural networks, robotics, biomedical engineering, electronics, electromagnetics, epidemiology, power systems and signal processing. This work may be also beneficial to inexperienced researchers and neophytes since it both summarizes the last PSO developments and outlines the best practices for the implementation of a customized algorithm from scratch.

It is straightforward that PSO has been widely adopted to interpret gravity, magnetic, SP and DC data, because of the simplicity of the forward modeling routine and of the implementation of the optimization scheme. A lot of literature works presents simplistic PSO applications to low “dimensional” geophysical problems (few tens of unknowns), deploying basic versions of the algorithm (e.g., constant accelerations, a single stopping criterion) with no uncertainty assessment of the final solution and limited use of computational resources. This review presented the state of the art and may pave the way for new possible directions of future research and encourage further developments in the theory and application. Future studies are highly recommended to speed up the computation by means of code parallelization and to openly discuss the obtained solutions by means of posterior analysis.

A possible direction for future work could be the solution of the 3-D EM inverse problem, which is highly challenging due to the strong nonlinearity of the forward model and the computational demand. Moreover, new criteria can be studied for the implementation of PSO where the solution of the problem is driven or constrained by additional information such as geological and stratigraphic data. A new possible research trend should be the coexistence of the local and global approaches to solve the inverse problem in order to consider two families of possible solutions, given that global search methods are going to be positively accepted in spite of the skeptical view of the past.
